# Pd Doped on TCH@SBA-15 Nanocomposites: Fabrication and Application as a New Organometallic Catalyst in the Three-Component Synthesis of *N*-Benzo-imidazo- or -thiazole-1,3-thiazolidinones

**DOI:** 10.3389/fchem.2021.723207

**Published:** 2021-10-04

**Authors:** Mehdi Kalhor, Akbar Dadras

**Affiliations:** Department of Chemistry, Payame Noor University, Tehran, Iran

**Keywords:** Pd@SBA-15, organometallic nanocatalyst, thiocarbohydrazide, three-component reaction, 2-aminobenzimidazole, 2-aminobenzothiazole, 1,3-thiazolidinone

## Abstract

In this study, Pd(II)/TCH@SBA-15 nanocomposites were synthesized by the grafting of 3-chloropropyltriethoxysilane and thiocarbohydrazide on SBA-15 and subsequent deposition of palladium acetates through the ligand–metal coordination method. The structure and morphology of this nanoporous nanocomposite was thoroughly identified by Fourier transform infrared spectroscopy, field emission scanning electron microscopy, transmission electron microscopy, energy-dispersive X-ray spectroscopy, X-ray diffraction, thermogravimetric analysis, atomic absorption spectroscopy, and Brunauer–Emmett–Teller instrumental analyses. Furthermore, the catalytic activity of this nanocomposite was investigated in the three-component synthesis of 3-benzimidazolyl or benzothiazoleyl-1,3-thiazolidin-4-ones *via* a reaction of 2-aminobenzimidazole or 2-aminobenzothiazole, aromatic aldehydes, and thioglycolic acid in an acetone–H_2_O mixture under green conditions. The Pd/TCH@SBA-15 nanocatalyst is demonstrated to exhibit a high catalyzing activity in the three-component reaction of the synthesis of *N*-heterocyclic thiazolidinones with good to excellent yields. One of the advantages of the suggested method is the direct application of the thiocarbohydrazide ligand to stabilize Pd nanoparticles through formation of a stable ring complex without creating an additional Schiff base step. Moreover, this organometallic nanocatalyst can be recycled several times with no notable leaching or loss of performance.

## Introduction

Today, continuous efforts are made to place organometallic functional groups on heterogeneous supports and apply them in catalytic reactions. Supported catalysts have both homogeneous and heterogeneous catalytic benefits (Wan and Zhao, 2007; Muñoz-Batista et al., 2018; Rodríguez-Padrón et al., 2019; Rodríguez-Padrón et al., 2019; Tamoradi et al., 2020). Among the suitable supports, SBA-15 silicate has received considerable attention due to its desirable properties such as regular channel nanostructures, a pore diameter in the range of 2–30 nm, a high surface-to-volume ratio, suitable selectivity, high thermal and mechanical stability, and adjustable surface chemistry (Zhao et al., 1998; Fulvio et al., 2005; Ostovar et al., 2018; Rajabi et al., 2019). In addition, the ability to design, manipulate, and modify the surface of SBA-15 by organic and inorganic materials due to large amounts of surface hydroxyl groups, which is a key factor for its application in catalytic processes, has led to special attention to these porous structures (Feng et al., 2010; Bhuyan et al., 2015; Ostovar et al., 2018). One of the convenient procedures for applying SBA-15 nanoparticles as a catalyst is to anchor the desired functional groups on high surface mesoporous silica. In these cases, a covalent interaction could be established between the organic functional group and the silica framework, which prevents leaching of the active sites into the reaction mixture after repeated use. Therefore, heterogeneous catalysts with active metal centers supported on SBA-15–modified nanoparticles could be an ideal option in catalytic systems (Karimi et al., 2006).

Pd species are one of the most versatile catalysts used in modern organic synthesis and have been widely used in a significant number of synthetic transformations (Kim et al., 2012; Veisi et al., 2019) and cross-couplings and related reactions, such as Heck, Suzuki, Stille, and Sonogashira types (Calo et al., 2005; Tamami et al., 2011; Biffis et al., 2018; Veisi et al., 2019; Tamoradi et al., 2020; Veisi et al., 2020). So far, several reviews have been reported on the role of Pd as a metal catalyst with or without support in classical and modern organic reactions (Lamblin et al., 2010; Molnár, 2011; Biffis et al., 2018; Veisi et al., 2020). Pd salts or complexes, preformed or produced *in situ* upon addition of a ligand, are commonly utilized as sources of palladium for these reactions. It is known that well-dispersed Pd catalysts immobilized on suitable supports, such as the SBA-15, can enhance their catalytic activity. Finally, the development of a new and efficient approach for the stabilization of Pd nanoparticles onto the SBA-15 surface as organometallic materials is still needed for catalytic organic transformation.

1,3-Thiazolidine-4-ones with sulfur and nitrogen atoms and a carbonyl group in a 5-membered ring belong to a large family of thiazoles, which are of special importance and attention due to their extensive activities in the biological, pharmaceutical, and agricultural fields (Cunico et al., 2008; Mobinikhaledi et al., 2010; Jain et al., 2012; Zhang et al., 2020). The basic component of thiazolidinone has been used in the structure of various important drugs, such as thiazolidomycin (antibiotic), ralitoline (antiepileptic), rosiglitazone (antidiabetic), and etozoline (loop diuretic). This widespread application highlights the importance of access to effective synthetic pathways for the production of thiazolidine-4-one compounds. The general method of synthesis can be either a one-pot or a two-step process of an amine with aromatic aldehydes and thioglycolic acid, under traditional hard conditions, or with the use of heterogeneous and homogenous catalysts (Srivastava et al., 2001; Kumar et al., 2013; Thakare et al., 2014; Jadhav et al., 2015; Chate et al., 2016; Harale et al., 2016; Pang et al., 2016; Safaei-Ghomi et al., 2016; Safaei-Ghomi et al., 2016; Thakare et al., 2016; Kalhor et al., 2017; Kalhor et al., 2018; Kalhor and Banibairami, 2020). However, despite reports of good progress in the synthesis of these valuable compounds, there are still some limitations to these methods and research and development to achieve new, more efficient, and greener synthetic strategies is useful and in demand.

**SCHEME 1:**
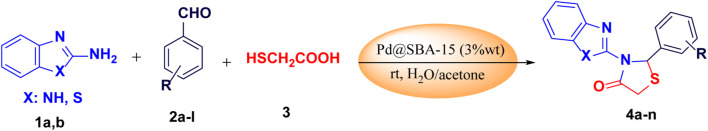
A synthetic method for *N*-heterocyclic-1,3–thiazolidinones.

Considering the above points in our ongoing research on the synthesis of organometallic nanocatalysts (Kalhor et al., 2019; Kalhor et al., 2020), we report herein a simple method for the preparation of stable and active Pd supported on TCH@SBA-15 mesoporous composites as a nanocatalyst for the three-component synthesis of 2-aryl-*N*-benzo-imidazo- or -thiazole-1,3-thiazolidinones *via* condensation of the aldehyde derivatives 2-aminobenzimidazole or benzothiazole and thioglycolic acid under green conditions (Scheme 1).

## Experimental Methods

### Materials and Apparatuses

Chemical compounds were purchased from Sigma–Aldrich and Merck companies with a commercial grade and used as received without further purification. Melting points were determined in open capillaries using an electrothermal digital melting point apparatus and were uncorrected. Fourier transform infrared (FT-IR) spectra were recorded on a JASCO 4200-A spectrometer with KBr pellets. ^1^HNMR and ^13^CNMR spectra were recorded on a Bruker spectrometer (300–500 MHz) using DMSO-*d*_6_ as a solvent and Me_4_Si as an interior standard. The morphology of the functionalized SBA-15 was investigated using a Leica Cambridge S 360 scanning electron microscope (scanning electron microscopy (SEM) and field emission scanning electron microscopy (FE-SEM)). Transmission electron microscopy (TEM) images were recorded on a Philips CM10 microscope with an acceleration voltage of 100 kV. N_2_ adsorption–desorption isotherms of SBA-15 nanocomposites were measured at the temperature of liquid nitrogen using a Micromeritics system (made in the United States). The Brunauer–Emmett–Teller surface area of the nanoparticles was calculated using the BET method.

### The Typical Procedure for the Synthesis of Pd/TCH-pr@SBA-15 Nanocatalysts

Chloropropyl-functionalized SBA (Pr-Cl@SBA-15) was prepared according to the literature by Kalhor et al. (2019). Then, for the synthesis of TCH/Pr-Cl@SBA-15 nanohybrids, in a 100 ml round-bottom flask, 1.2 g (11.3 mmol) of TCH was dissolved in 18 ml of acetonitrile and stirred for 1 h. Next, 1.17 g (6.67 mmol) of KI and 1.2 g of SBA-15-Cl were added to the reaction mixture and refluxed for 15 h. The solvent was removed, and the mixture was collected using a centrifuge. The resultant solid mixture was stirred in 50 ml distilled water to remove the excipients or unreacted particles, separated by vacuum filtration using a Buchner funnel, and dried in an oven at 50 °C for 12 h. For the complex formation of Pd(II) ions on functionalized SBA-15, 0.15 g (0.67 mmol) of palladium acetate was dissolved in 10 ml acetone, and 1.30 g of SBA-15-TCH was added to the solution and stirred at room temperature for 3 h. The mixture was filtered, washed with acetone and THF, and dried in an oven at 50 °C for 3 h to obtain the Pd/TCH-pr@SBA-15.

### General Procedure for the Preparation of 1,3-Thiazolidin-4-ones

A mixture of 2-aminobenzimidazole or 2*-*aminobenzothiazole (1 mmol), an aldehyde (1 mmol), thioglycolic acid (1 mmol), and 3 wt% of the Pd/TCH-pr@SBA-15 nanocatalyst (0.004 g) in 5 ml H_2_O/acetone (1:1) was stirred at room temperature. After completion of the organic reaction [thin layer chromatography monitoring using hexane and ethyl acetate (2:1) as eluents], the nanocatalyst was intercepted by filtration and the filtrate was added to 10 ml of cold water. The precipitate was filtered out and washed with a cold ethanol–water mixture. Most of the desired products were obtained on a pure basis; however, for further purity, these can be recrystallized from the water–ethanol mixture. All the 1,3-thiazolidin-4-one products were known and identified by spectroscopic data and by comparing their melting points with literature values. Spectroscopic data for some 1,3-thiazolidin-4-ones are given in the following sections.

### Spectroscopic Data for the Selected Compounds

#### 3-(1*H*-Benzo[d]imidazol-2-yl)-2-(2-chlorophenyl)thiazolidin-4-one (4f)

FT-IR (KBr) (*ν*_max_): 3444, 3349 (NH), 2925 (C-H), 1683 (C=O), 1535 (C=N), 1447, 1303, 1269 (C=C), 1170 (C-N), 743, and 647 (C-S-C) cm^−1^; ^1^H-NMR (500 MHz, DMSO-*d*_*6*_) *δ*_*H*_: 12.52 (1H, s, NH), 7.56 (2H, t, *J* = 7.60 Hz, H-Ar), 7.39 (1H, d, *J* = 7.90 Hz, H-Ar), 7.33 (1H, t, *J* = 7.60 Hz, H-Ar), 7.27 (1H, t, *J* = 7.55 Hz, H-Ar), 7.14–7.07 (3H, m, H-Ar), 6.85 (1H, s, CH), 4.14 (1H, d, *J* = 16.45 Hz, SCH-diastereotopic), and 4.01 (1H, d, *J* = 16.50 Hz, SCH-diastereotopic) ppm; ^13^C-NMR (125 MHz, DMSO-*d*_6_) *δ*_C_: 172.2, 144.7, 140.2, 138.3, 133.3, 131.5, 130.5, 129.9, 128.1, 125.2, 122.2, 122.0, 118.2, 112.4, 59.5, and 32.1 ppm; MS (m/z, %): 329 (M^+^, 10), 294 (100), and 220 (94).

#### 3-(Benzo[*d*]thiazol-2-yl)-2-(4-bromophenyl)thiazolidin-4-one (4m)

FT-IR (KBr) (*ν*_max_): 1690 (C=O), 1617, 1537 (C=N), 1467, 1369, 1274 (C=C), 1228 (C-N), and 658 (C-S-C) cm^−1^; ^1^H-NMR (300 MHz, DMSO-*d*_*6*_) *δ*_H_: 8.02 (1H, d, *J* = 7.74 Hz, H-Ar), 7.66 (1H, d, *J* = 8.75 Hz, H-Ar), 7.53 (2H, d, *J* = 8.41 Hz, H-Ar), 7.41–7.30 (4H, m, H-Ar), 6.89 (1H, s, CH), 4.31 (1H, d, *J* = 16.73 Hz, SCH_2_), and 4.05 (1H, d, *J* = 16.76 Hz, SCH_2_) ppm; ^13^C-NMR (75 MHz, DMSO-*d*_6_) *δ*_C_: 31.8, 62.1, 121.0, 121.2, 121.9, 124.4, 126.3, 127.7, 131.2, 131.5, 140.6, 147.5, 155.9, and 171.6 ppm; MS (m/z, %): 392.4 (M^+^, 58.4), 350.4 (42.5), 317.4 (83.2), and 135.5 (100).

#### 3-(Benzo[*d*]thiazol-2-yl)-2-(4-methoxyphenyl)thiazolidin-4-one (4n)

FT-IR (KBr) (*ν*_max_): 1690 (C=O), 1617, 1537 (C=N), 1467, 1369, 1274 (C=C), 1228 (C-N), and 658 (C-S-C) cm^−1^; ^1^H-NMR (300 MHz, DMSO-*d*_*6*_) *δ*_H_: : 8.02 (1H, d, *J* = 7.69 Hz, H-Ar), 7.68 (1H, d, *J* = 7.97 Hz, H-Ar), 7.42–7.29 (4H, m, H-Ar), 6.88 (2H, d, *J* = 7.51 Hz, H-Ar), 6.85 (1H, s, CH), 4.31 (1H, d, *J* = 16.79 Hz, SCH_2_), 4.04 (1H, d, *J* = 16.81 Hz, SCH_2_), and 3.69 (3H, s, OMe) ppm; ^13^C-NMR (75 MHz, DMSO-*d*_6_) *δ*_C_: 31.9, 55.0, 62.5, 113.9, 121.2, 121.8, 124.3, 126.3, 127.0, 131.2, 132.9, 147.6, 155.9, 158.9, and 171.6 ppm; MS (m/z, %): 342.6 (M^+^, 99.9), 300.6 (71.6), 267.6 (80.5), 208.5 (68.8), and 135.5 (100).

## Results and Discussion

### Preparation and Characterization of the Pd/TCH-Pr@SBA-15 Nanocatalyst

**SCHEME 2:**
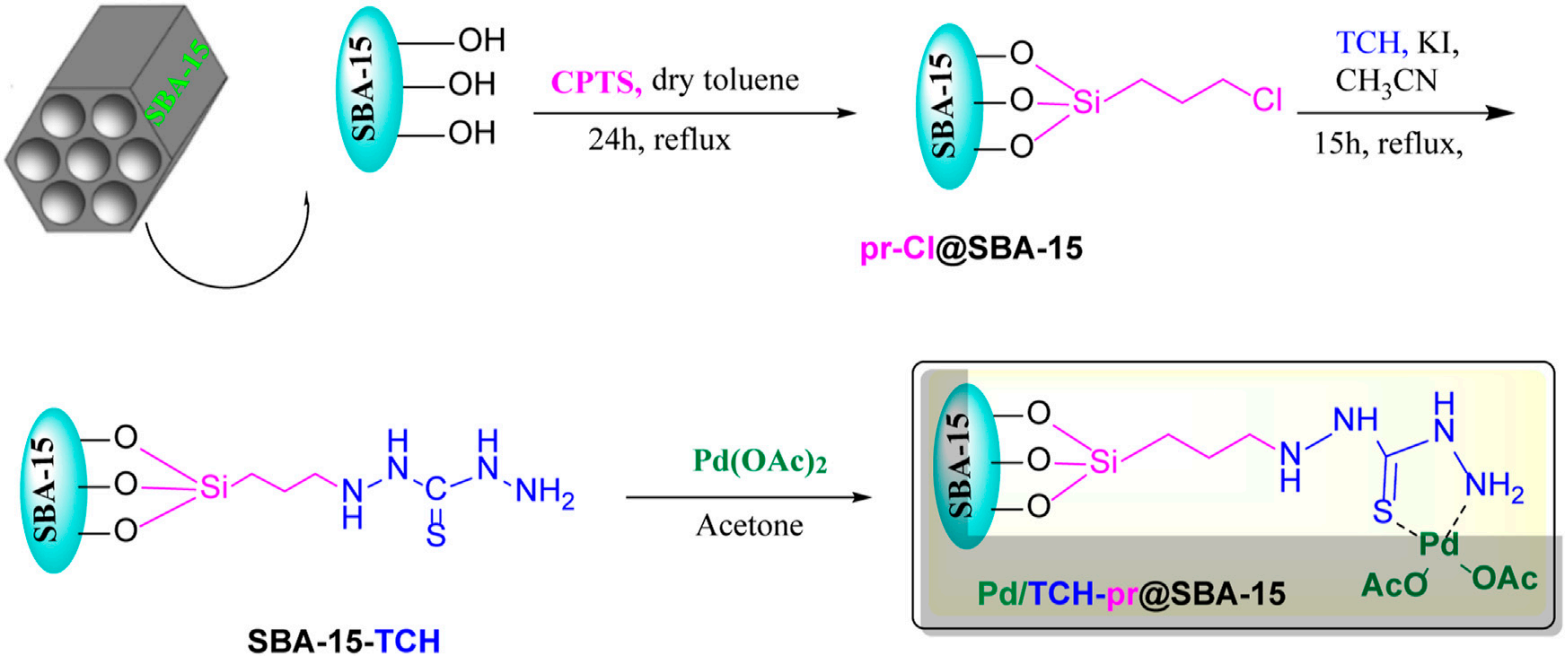
A schematic diagram for the synthesis of the Pd/TCH@SBA-15 nanocatalyst.

The organometallic nanohybrid composite Pd/TCH-pr@SBA-15 was designed and fabricated according to Scheme 2. SBA-15 was functionalized with the organosilane precursor 3-chloropropyltriethoxysilane according to the reported method by Kalhor et al. (2019). Then, this chloropropyl-grafted SBA-15 was refluxed with a TCH ligand followed by complexation with Pd(OAc)_2_ to afford the supported Pd catalyst on the SBA-15 nanoparticles. Since the investigation of the composition, structure, and morphology of nanocatalysts is an important parameter in the prediction of their catalytic behavior, the structure of the Pd/TCH-pr@SBA-15 nanocomposite was first studied using different techniques and is discussed in the following.

**FIGURE 1:**
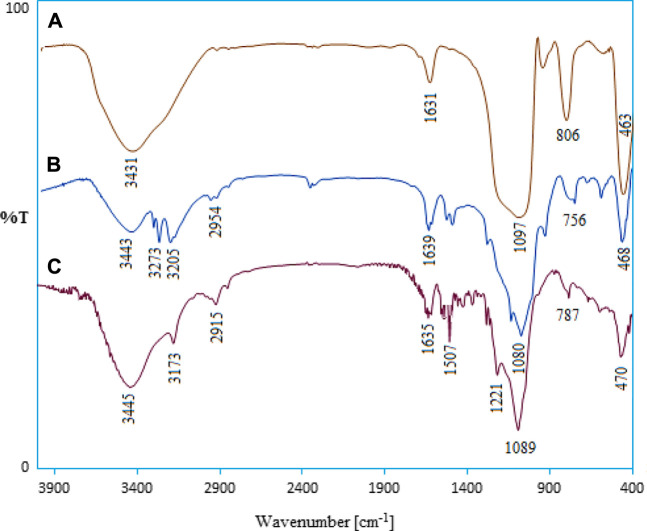
FT-IR spectra of **(A)** SBA-15, **(B)** SBA-15-TCH, and **(C)** Pd/TCH@SBA-15.

In order to show the nanostructure functional groups, the crude SBA-15 FT-IR spectra and its modified spectra were analyzed. Figure 1A demonstrates the spectrum of SBA-15 nanoparticles. The broad absorption band appearing at 3431 cm^−1^ is due to the stretching vibration of Si-OH functional groups. Also, absorption bands at 1631 and 1097 cm^−1^ are specification of water bonded to the silica backbone and asymmetric stretching of Si-O-Si, respectively (Safaei-Ghomi et al., 2016). The appearance of signals in the 3273 and 3205 cm^−1^ regions (NHNH_2_ group) in Figure 1B confirms the presence of thiocarbohydrazide in the nanostructure. The stretching vibrations C=S are characterized by two index wavenumbers (1290 and 931 cm^−1^) in TCH, respectively. The peak in the 2954 cm^−1^ region indicates the binding of CH_2_ groups to the silicate nanostructure. From the comparison of the last two spectra, given the identical chemical nature of the two compounds, the apparent similarity of the spectra is natural and predictable. However, in the spectrum of the organometallic nanostructure, changes in wavenumbers of amine (NH_2_) and thione (C=S) functional groups can be observed, which is related to the coordination interactions of Pd(II) ions with nitrogen and sulfur atoms in the TCH section (Burns, 1968; Braibanti et al., 1969; Alizadeh et al., 2013). Overall, the comparison of the infrared spectra, as expected, confirms the immobilization of the desired organometallic functional groups on the surface of SBA-15.

**FIGURE 2:**
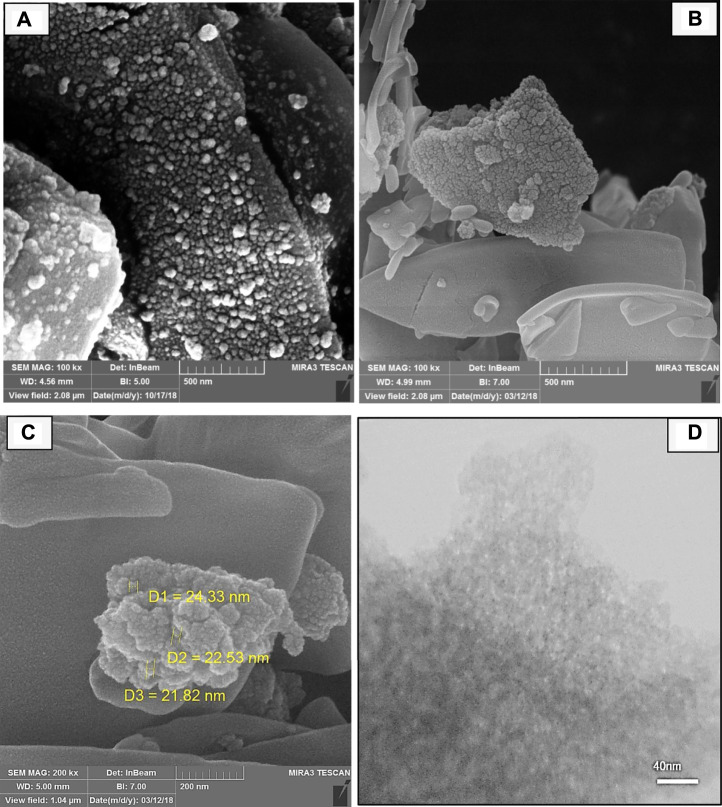
FE-SEM images of **(A)** SBA-15-TCH and **(B, C)** Pd/TCH-pr@SBA-15. **(D)** Their TEM micrograph.

FE-SEM was used to investigate the surface morphology of SBA-15-TCH and Pd/TCH@SBA-15 composite nanomaterials, and the corresponding images are shown in Figure 2. As observed, SBA-15-TCH has a crystalline structure in the form of a spherical shape (Figure 2A). Furthermore, the structure of the Pd/TCH@SBA-15 nanocomposite is clearly demonstrated in Figures 2B,C. The original structure of SBA-15 remained unchanged following functionalization. According to Figure 2C, the particle size is in the range of 21–24 nm. The TEM micrographs in Figure 2C illustrate regular mesoporous channels of silica after modification.

**FIGURE 3:**
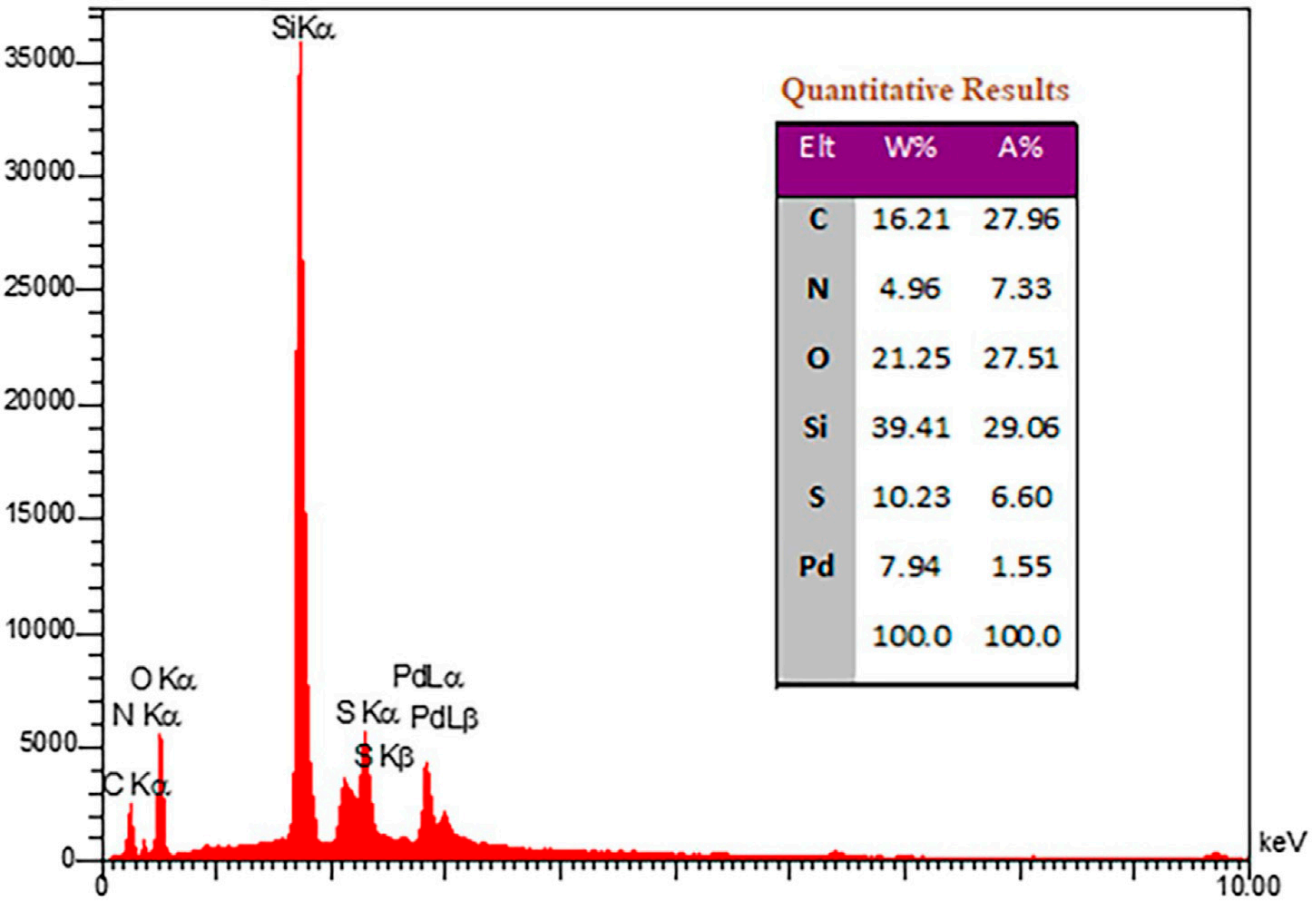
The EDX diagram of Pd/TCH-pr@SBA-15.

The energy-dispersive X-ray spectroscopy (EDX) of the prepared organometallic nanosystem is presented in Figure 3. The signals corresponding to C, N, S, O, Si, and Pd elements as well as the quantitative results of EDX confirm the presence of Pd(II) and propyl-TCH moieties in the Pd/TCH-pr@SBA-15 nanostructure. Moreover, the Pd content of the Pd/TCH-pr@SBA-15 estimated by atomic absorption spectroscopy is 22% wt/wt%.

**FIGURE 4:**
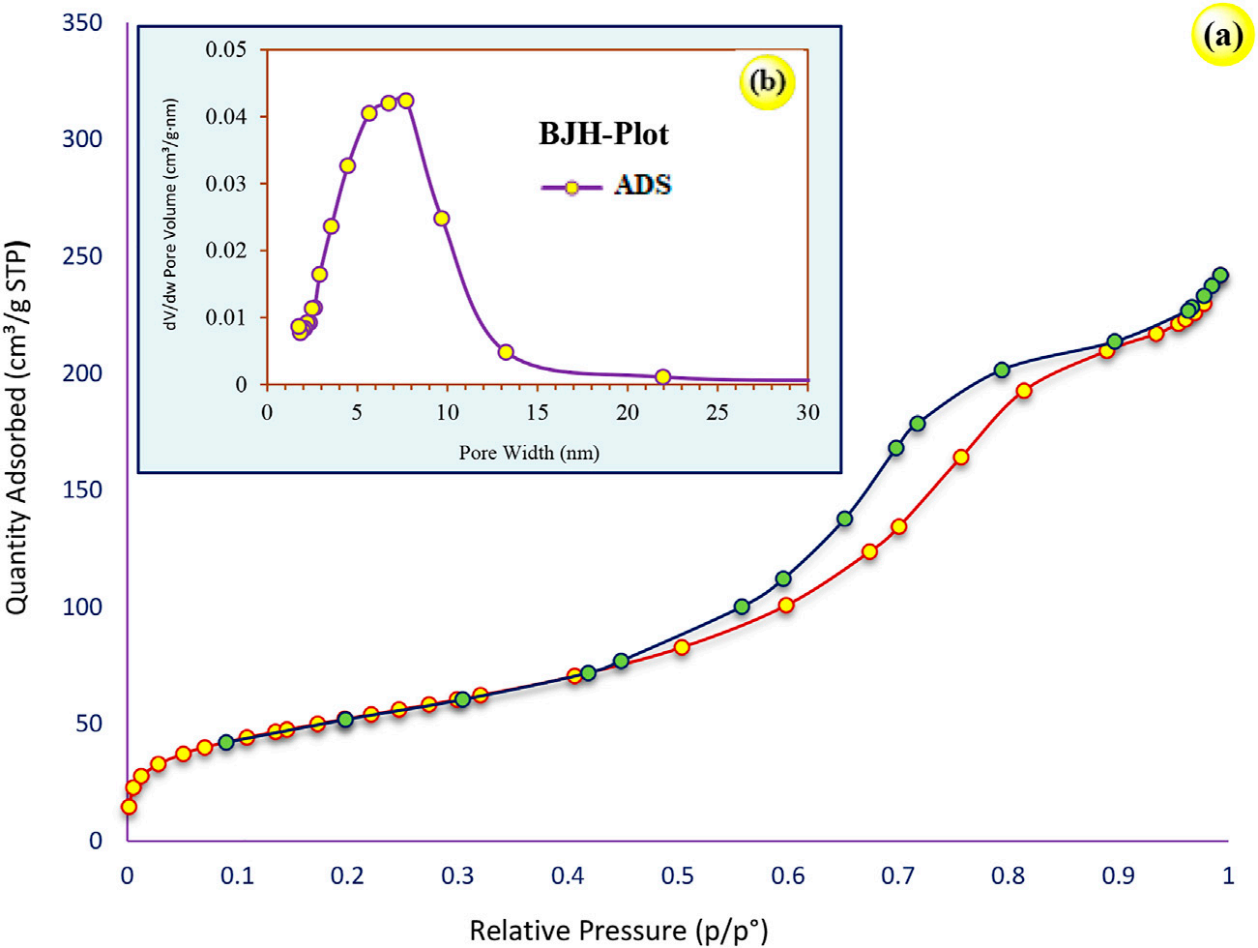
**(A)** Adsorption–desorption N_2_ isotherms and **(B)** pore size distribution of Pd/TCH-pr@SBA-15.

Figure 4A shows the N_2_ adsorption–desorption isotherms of Pd/TCH-pr@SBA-15 nanocomposites. As observed, this structure shows type I isotherm, representing microporous and mesoporous frameworks. According to Figure 4B, the N_2_ adsorption–desorption diagram of Pd/TCH-pr@SBA-15 shows type IV isotherms with a sharp capillary condensation step at high relative pressure and an H1 hysteresis loop in the 0.4–0.9 p/p^0^ range, which reveals the presence of large channel-like pore structures in a narrow range of size, based on the IUPAC classification (Safaei-Ghomi and Bakhtiari, 2018). Also, by the shape of its hysteresis, it can be seen that Pd/TCH-pr@SBA-15 has cylindrical pores and the initial nanostructure after functionalization is still retained (Sánchez-Velandia and Villa, 2019).

Moreover, the BJH pore size distribution diagram in Figure 4B shows that the major width of the cavities is in the range of 2–21 nm and cavities are microporous and mesoporous (Sarkar et al., 2009).

The structural data of the SBA-15 and Pd/TCH-pr@SBA-15 nanoparticles are listed in Table 1. The BET surface area and pore volume of Pd/TCH@SBA-15 decrease owing to the functionalization steps of SBA-15 which may be due to the significant loading of the complex into the mesoporous channels of SBA-15 (Alizadeh et al., 2013). In addition, the average nanoparticle size is increased to 33 nm compared to that of SBA-15 (9 nm), which can confirm the grafting of organometallic functional groups on the substrate surface and the occupation of cavities.

**TABLE 1:** Structural and textural parameters of the SBA-15 and Pd/TCH@SBA-15 composites.

Sample	M^a^ (%)	S_BET_ ^b^ (m^2^.g^−1^)	*V*_BJH_ ^c^ (cm^3^.g^−1^)	D_BJH_ ^d^ (nm)	V_HKM_ ^e^ (cm^3^.g^−1^)	P_APS_ ^f^ (nm)
SBA-15	-	629.63	0.836	5.11	0.258	9.529
Pd/TCH-pr@SBA-15	22	184.33	0.382	7.09	0.077	31.482

a Initial percentage of copper ions.

b Specific surface area.

c Pore volume.

d Pore size (calculated from the adsorption branch).

e Maximum pore volume at p/p° = 0.172869793 (estimated using the Horvath–Kawazoe method).

f Average nanoparticle size (estimated using the Temkin method).

**FIGURE 5:**
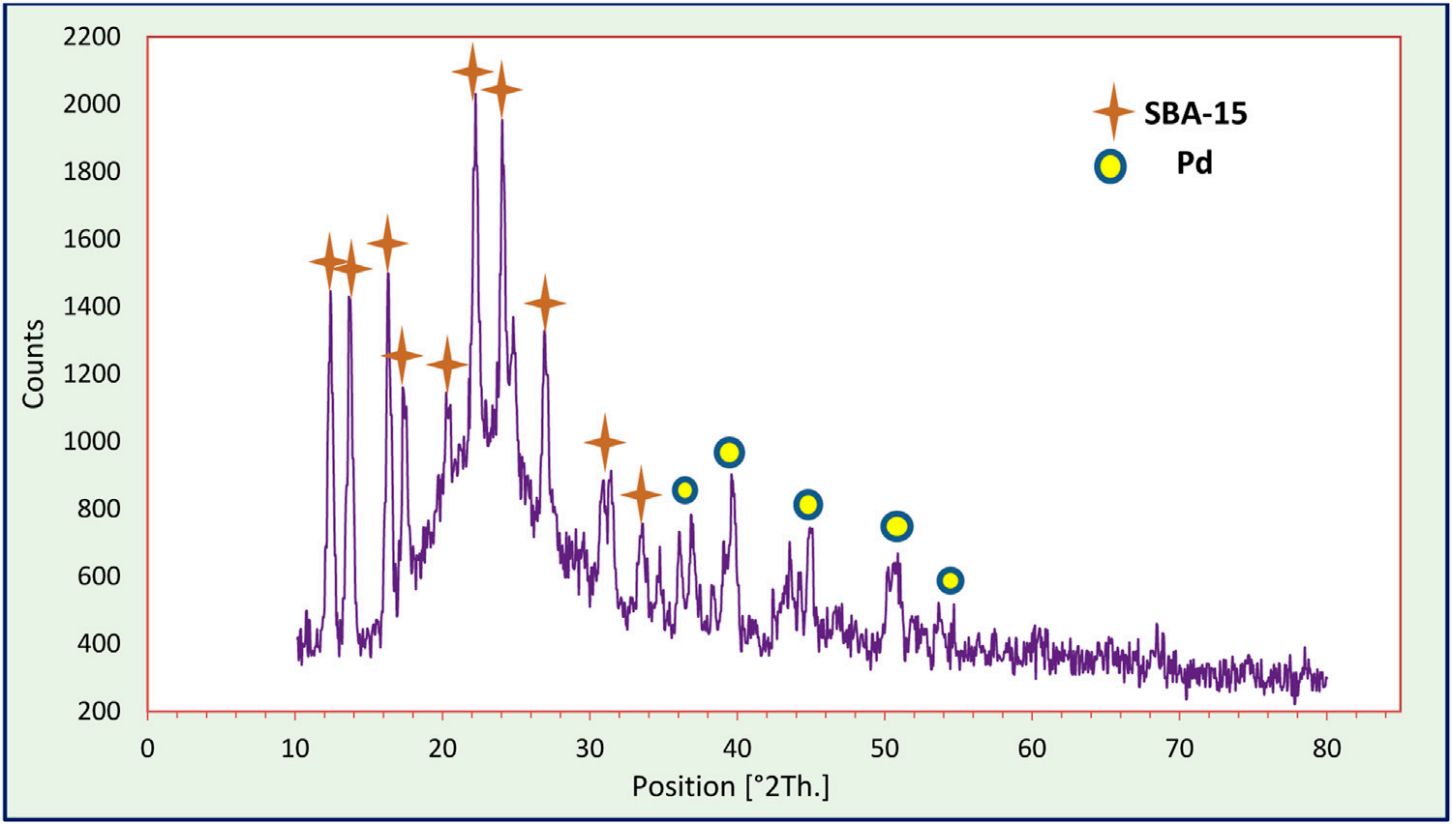
The XRD spectrum of the organometallic nanocatalyst Pd/TCH-pr@SBA-15.

The X-ray diffraction (XRD) pattern of Pd/TCH-pr@SBA-15 is shown in Figure 5. The XRD pattern of SBA-15 matches with the standard XRD data (JCDPS card no. 01-086-1561), in which the appearance of sharp diffraction peaks in it indicates a crystalline structure. The flattening of the peak at angles of 20°–25° (2*θ*) is due to the presence of amorphous silica phase in the SBA-15 structure, which corresponds to the mentioned JCPDS card number. The main peaks at 2*θ* values of 39.9° and 45.6° belong to crystal indexes of (111) and (200), respectively, which indicates the presence of palladium in the nanocomposite (Duan et al., 2017; Ulusal and Güzel, 2018). Also, the weak peaks at 2*θ* = 36.1°, 50.7°, and 54.7° related to crystal indexes of (101), (202), and (112), respectively, according to the standard XRD data (JCDPS card no. 43-1024), can indicate the presence of Pd^2+^ ions in the structure (Ganji et al., 2013; Payehghadr et al., 2019). The observation of these points indicates that the existence of an ordered two-dimensional (2D) hexagonal structure and also the framework of SBA-15 were not damaged after modification. The size of the Pd-TCH@SBA-15 nanostructure crystal determined from the XRD pattern using the Debye–Scherrer equation was 27.16 nm.

**FIGURE 6:**
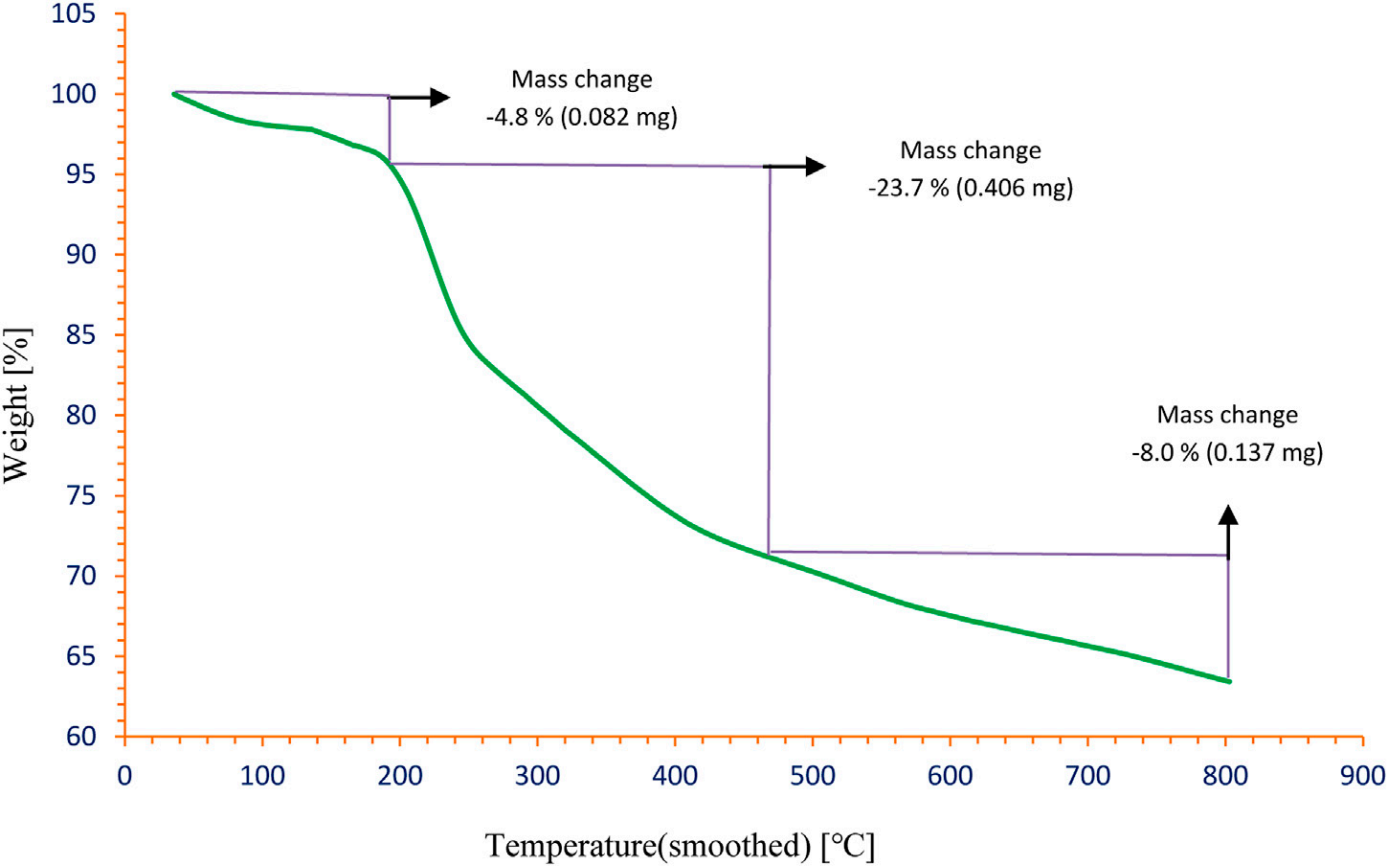
The TGA curve of Pd/TCH-pr@SBA-15.

Figure 6 shows the thermogravimetric analysis (TGA) curve of Pd/TCH-pr@SBA-15. As observed in the TGA curve, the weight loss of 4.8% (0.082 mg) in the first step in the temperature range of 50–190°C is due to physical and chemical desorption of water molecules. The second weight loss of 23.7% (0.406 mg) observed in the temperature range of 190–450°C is associated with the degradation of organic and complex (TCH-Pd) species. Thus, the SBA-15 surface is functionalized by organic groups. The final weight loss of 8.0% (0.137 mg) in the temperature range of 450–800°C may be due to the change in the silica structure (Liu et al., 2010).

### Catalytic Activities of Pd/TCH@SBA-15 in the Solvent-Free Synthesis of 3-Benzimidazolyl- or Benzothiazoleyl- 1,3-thiazolidin-4-ones

In the preliminary experiment to find optimal conditions for the synthesis of 2-aryl-3-benzimidazolyl or benzthiazoleyl-1,3-thiazolidin-4-ones, we used 3-nitrobenzaldehyde, 2-aminobenzimidazole, and thioglycolic acid as starting materials in the presence of Pd/TCH-pr@SBA-15 as a new nanocatalyst for a model reaction. To achieve optimal conditions, the desired reaction was carried out with different solvents and catalyst amounts as a green process. It is shown in Table 2 that when 3 wt% of Pd/TCH-pr@SBA-15 was used, 95% of the desired product was formed at 25 °C in acetone/H_2_O (1:1) (Table 2, entry 7). Moreover, it was observed that this organic reaction did not improve in the presence of Pd(OAc)_2,_ SBA-15, and TCH@SBA-15 (Table 2, entries 14–16, respectively). Also, a glancing look at Table 2 exhibits that the reaction yields increase in the aprotic–protic polar solvent mixture (water/acetone). On the contrary, without the nanocatalyst, the reaction did not proceed (Table 2, entry 17).

**TABLE 2:** Optimization of the reaction conditions for the synthesis of 3-(1*H*-benzo[d]imidazol-2-yl)-2-(3-nitrophenyl)thiazolidin-4-one for the model reaction.
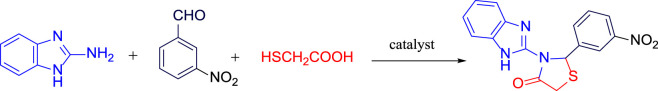

Entry	Catalyst loading (wt/wt%)	Solvent	Time (min)/temperature (°C)	Yield (%)^a^
1	5	EtOH	30/25	85
2	7	EtOH	25/25	77
3	3	EtOH	30/25	90
4	1.5	EtOH	45/25	67
5	3	EtOH/H_2_O (1:1)	45/25	72
6	3	Acetone	20/25	90
**7**	**3**	**Acetone/H**_**2**_ **O (1:1)**	**20**/25	**95**
8	5	Acetone/H_2_O (1:1)	20/25	95
9	3	H_2_O	20/25	-
10	3	H_2_O	20/100	45
11	3	MeOH	20/25	73
12	3	CHCl_3_	20/25	trace
13	3	MeCN	20/25	67
14	3 (Pd(OAc)_2_)	Acetone/H_2_O (1:1)	20/25	55
15	3 (SBA-15)	Acetone/H_2_O (1:1)	20/25	trace
16	3 (TCH@SBA-15)	Acetone/H_2_O (1:1)	20/25	30
17	-	Acetone/H_2_O (1:1)	60/25	trace

Bold values are for the optimal reaction conditions.

a Isolated yield.

Under the optimized conditions of the model reaction, a study on a variety of aldehyde derivatives was carried out, and the representative results are presented in Table 3. Different aryl aldehydes were transformed into corresponding 3-benzimidazolyl or benzthiazoleyl-1,3-thiazolidin-4-ones, with good to excellent yields.

**TABLE 3:** Synthesis of products **4a–n** in the presence of 3 wt% of Pd/TCH-pr@SBA-15 and in an acetone/H_2_O mixture at room temperature.

Entry	Ar-CHO	Product	Time (min)	M.p. (^o^C)	Yield (%)^a^
1	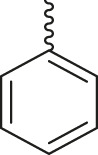	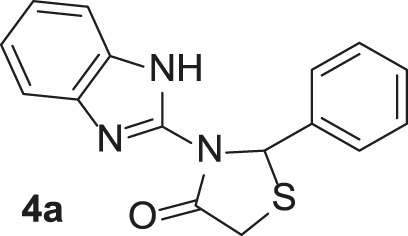	22	208–210	88
				(209–210)^b^	
2	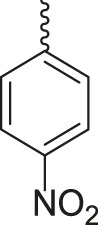	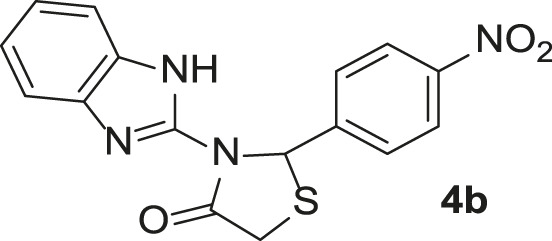	15	208–210	97
				(208–210)	
3	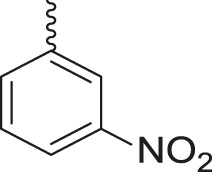	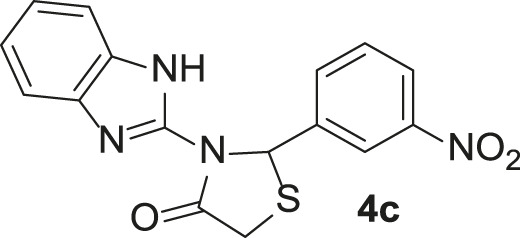	20	147	95
				(147–148)	
4	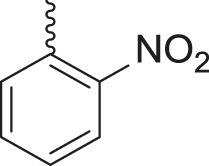	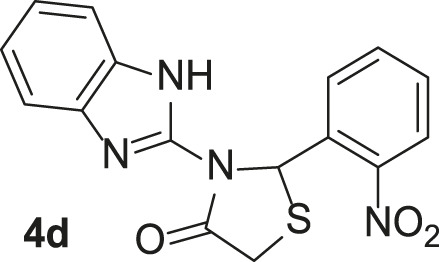	18	266–267	92
				(265–267)	
5	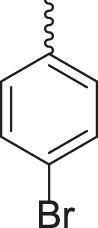	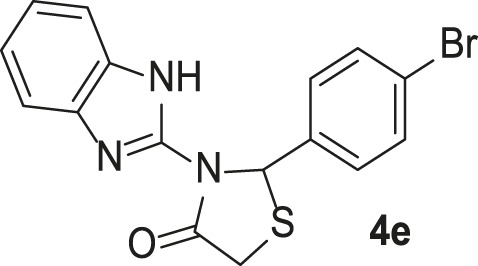	20	245	94
				(244–246)	
6	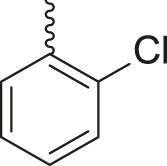	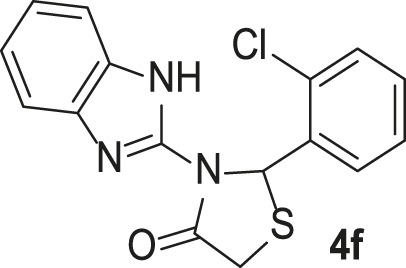	18	220–221	91
				(221–223)	
7	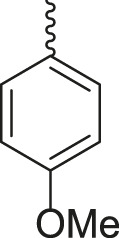	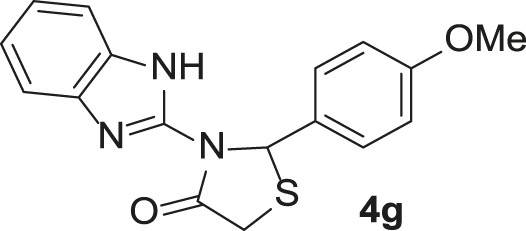	23	216–217	87
				(216)	
8	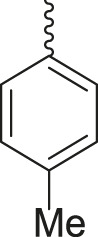	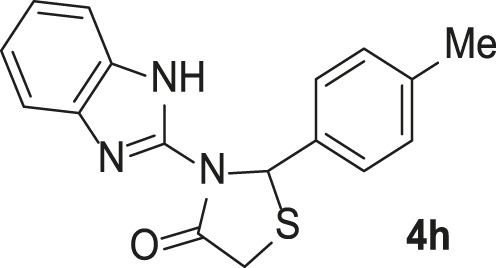	24	219	86
				(220)	
9	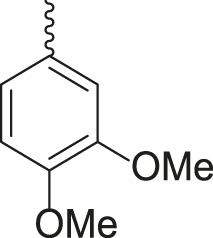	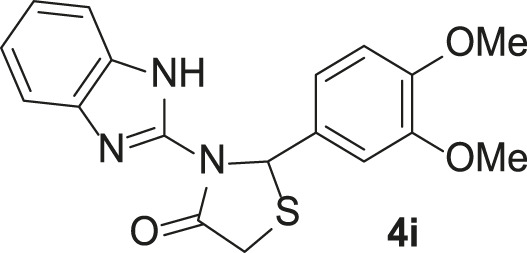	22	167–168	91
				(167–169)	
10	**2b**	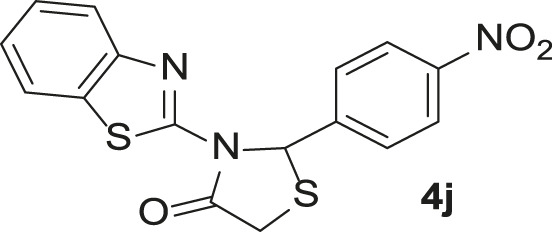	15	176–178	98
				(176)	
11	**2c**	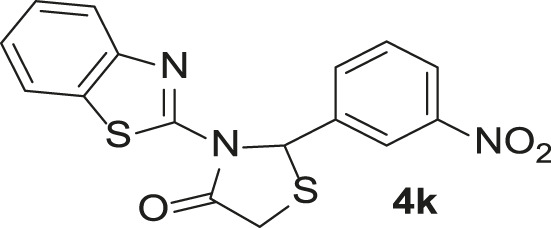	18	170–171	96
				(171–172)	
12	**2h**	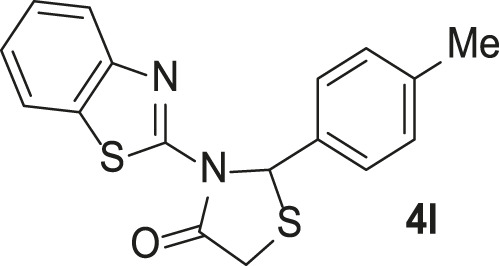	22	192	89
				(194–195)	
13	**2e**	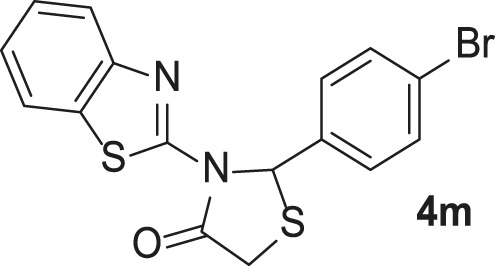	19	163–164	95
				(161–162)	
14	**2g**	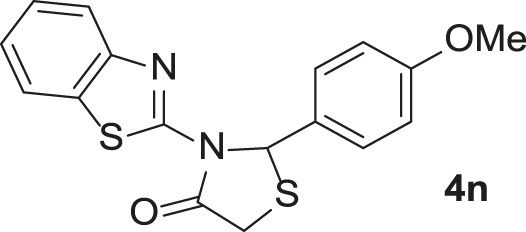	21	176	92
				(177–178)	

The bold values are for the aldehyde derivatives code.

a Isolated yield.

b Melting points in parentheses are reported in the literature by Kalhor and Banibairami (2020).

**SCHEME 3:**
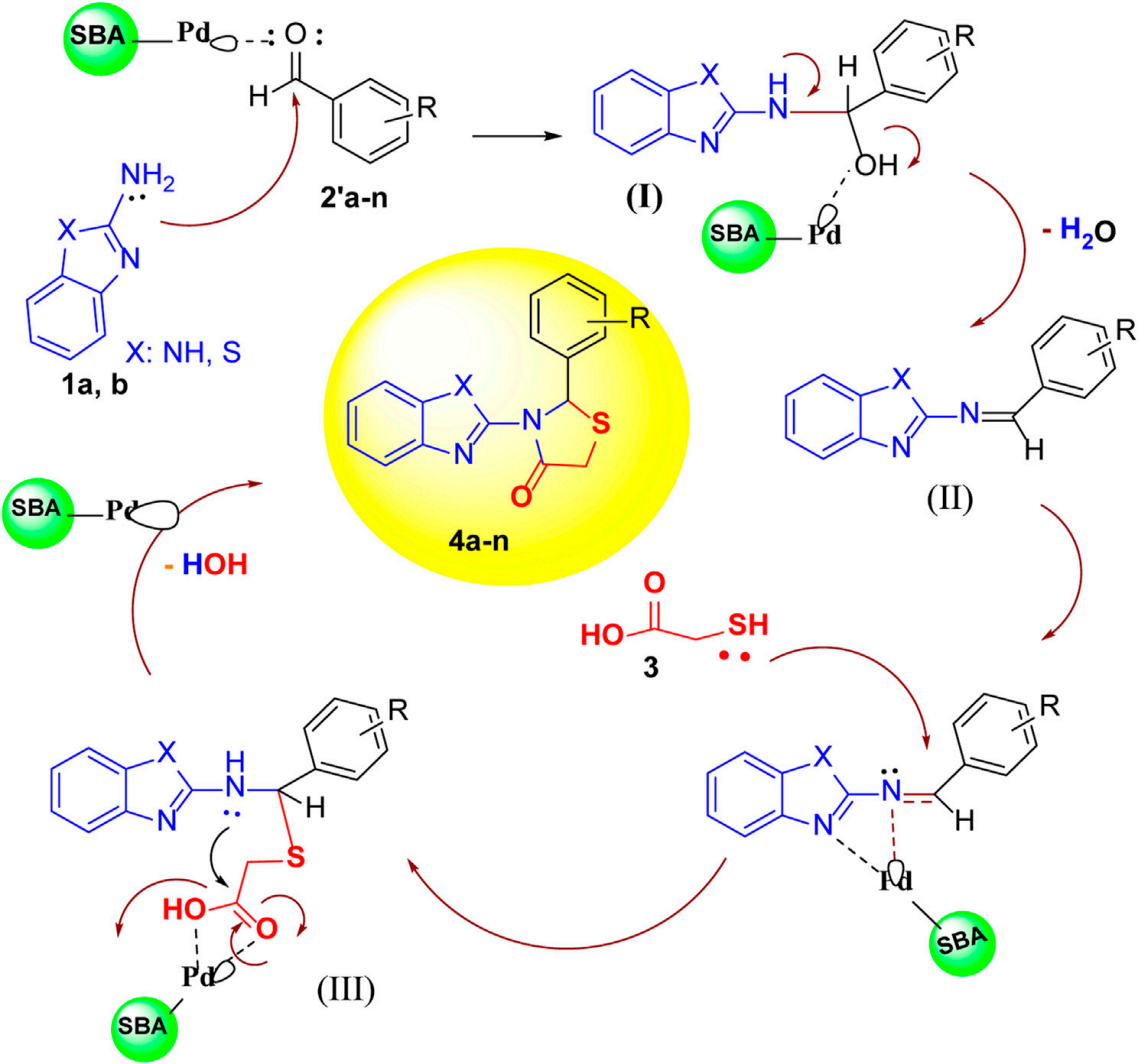
The proposed mechanism for the catalytic synthesis of compounds **4a–n** using Pd/TCH@SBA-15.

A proposed mechanism for the catalytic synthesis of 1,3-thiazolidin-4-ones by Pd-SBA-15 is shown in Scheme 3. First, the Pd/TCH-pr@SBA-15 nanocatalyst as a Lewis acid activates the carbonyl groups of aldehyde (**2a–n**). Then, the NH_2_ group of 2-aminobenzimidazole (or 2*-*aminobenzothiazole) as a nucleophile attacks the activated carbonyl to afford the intermediate **I** that is followed by the catalytic oxidation process and removal of a H_2_O molecule to form intermediate **II.** The Schiff base **II** is a stable structure and can be separated from the reaction moiety. In the second catalytic activation stage, the nucleophilic attack of the thiol group of thioglycolic acid to imine takes place to form the intermediate **III**. Finally, after intermolecular nucleophilic attack and loss of the second water molecule, cyclization of the 1,3-thiazolidin-4-one products **4a–n** can be performed.

**FIGURE 7:**
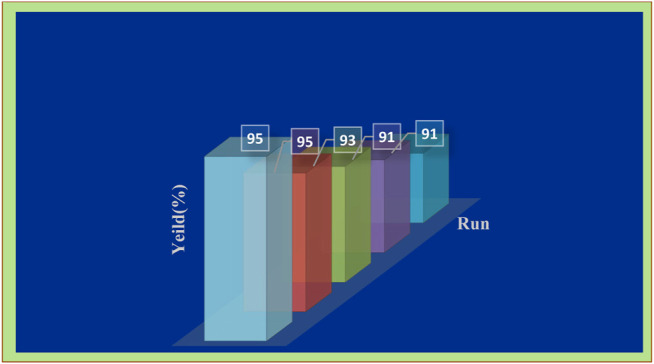
The study on the catalyst recycling activity in the synthesis of product **4c**.

The reusability of the heterogeneous catalyst was investigated for a model reaction under optimized conditions. After completion of the catalytic process, the Pd/TCH-pr@SBA-15 composite was easily separated by simple filtration from the reaction mixture. It was washed with ethanol several times, dried in an oven at 70 °C for 60 min, and then reused in the next reaction. The results of recyclability of the Pd/TCH-pr@SBA-15 composite are shown in Figure 7. No significant reduction in the catalyst activity was observed after five times. Therefore, the doped -(CH_2_)_3_-TCH-Pd composite on SBA-15 as an applicable support is found to be a superior efficient catalytic organometallic nanosystem for this reaction.

**FIGURE 8:**
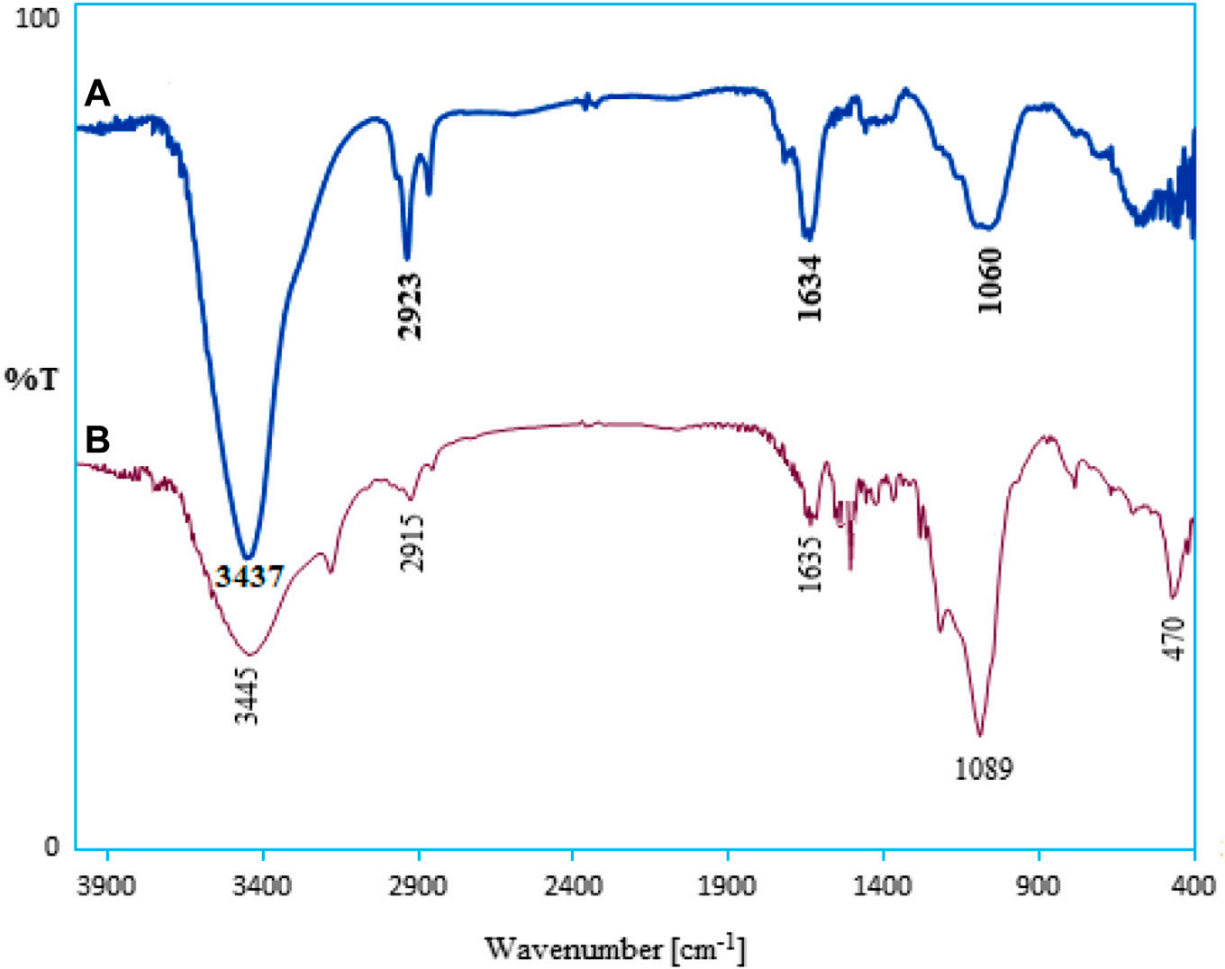
FT-IR spectra of the **(A)** fresh catalyst and **(B)** recovered Pd/TCH@SBA-15 catalyst after 5 cycles.

The basic structure of the recycled catalyst was also affirmed with FT-IR spectra, and there was no difference in the FT-IR spectra of the fresh and recovered catalysts, approximately (Figure 8).

Also, the Pd content in the nanocatalyst after the fifth reaction cycle was marginally decreased to 21.7% wt/wt (this reduction can be due to the error in experimental chemical analysis). As a result, the heterogeneous nature of the nanocatalyst is confirmed in this reaction and no palladium leaching occurred.

A comparison of the catalytic activity of the Pd/TCH-pr@SBA-15 nanocomposite and other reported various catalysts for the synthesis of 1,3-thiazolidin-4-ones is listed in Table 4. A comparative look at Table 4 reveals that the nanocatalyst performance is better than that of other reported catalysts in terms of yield, amount of the catalyst, and reaction time.

**TABLE 4:** Comparison of the activity of various catalysts for the synthesis of 1,3-thiazolidin-4-ones.

Entry	Catalyst	Condition	Time (min)	Yield (%) (ref.)
1	DCC (60 mol%)	THF, RT	60	59–95 (Srivastava et al. (2001))
2	Pd NPs (10 mol%)	Solvent-free, 100 °C	60	71–90 (Harale et al. (2016))
3	Ni@zeolite-Y (10 wt%)	EtOH, RT	25–35	80–95 (Kalhor et al. (2018))
4	HClO_4_–SiO_2_ (47 wt%)	PhCH_3_, 100 °C	180–360	70–88 (Kumar et al. (2013))
5	Alum (10 mol%)	M. V. grind, RT	25–30	85–93 (Jadhav et al. (2015))
6	Silica gel (0.5 g)	THF, RT	240–420	77–96 (Thakare et al. (2014))
7	DIPEA (3 equiv)	PhCH_3_, reflux	180–240	65–85 (Chate et al. (2016))
8	CoFe_2_O_4_@SiO_2_/Pr-NH_2_ (0.7 mol%)	PhCH_3_, reflux	120–480	75–85 (Safaei-Ghomi et al. (2016))
9	Ni/SO_3_H@zeolite-Y (5 wt%)	H_2_O–acetone, RT	15–25	85–97 (Kalhor and Banibairami (2020))
10	Fe_3_O_4_@SiO_2_/APTPOSS (8 wt%)	Solvent-free, 60°C	30	90–94 (Safaei-Ghomi et al. (2016))
11	La(NO_3_)_3_ (10 mol%)	EtOH, RT	24	77–90 (Kalhor et al. (2017))
12	MCM-41@Si-L, CuSO_4_ (7 wt%)	PhCH_3_, 110 °C	720	77–99 (Pang et al. (2016))
13	Catalyst-free	H_2_O, RT	240–420	79–96 (Thakare et al. (2016))
**14**	Pd@SBA-15 (3 wt%)	H_2_O–acetone, RT	15–24	87–97 (**this work**)

## Conclusion

In this study, the SBA-15–supported TCH-palladium complex was successfully designed and characterized as a novel organometallic nanocatalyst for the three-component synthesis of 3-benzimidazolyl or benzothiazoleyl-1,3-thiazolidin-4-ones under green conditions. Some of the advantages of the present procedure include high product yields, short reaction time, easy purification process, application of nanotechnology in the catalytic process, recyclability, low cost, and nontoxicity of the catalyst. These benefits make the procedure more environmentally benign and a significant contribution towards green chemistry.

## Data Availability

The original contributions presented in the study are included in the article/Supplementary Material; further inquiries can be directed to the corresponding author.
