# Supplementary material for: Pd Doped on TCH@SBA-15 Nanocomposites: Fabrication and Application as a New Organometallic Catalyst in the Three-Component Synthesis of N-Benzo-imidazo- or -thiazole-1,3-thiazolidinones
Source: Front Chem. 2021 Oct 4;9:723207. doi: 10.3389/fchem.2021.723207 (PMC8524445; doi:10.3389/fchem.2021.723207)
Supplement: Supplementary file 1 [file DataSheet2.docx]

**Pd doped on nanocomposite TCH@SBA-15: fabrication and application as a new organometallic catalyst in three-component synthesis of *N*-benzo- imidazo- or -thiazole-1,3-thiazolidinones**

**Synthesis of Nano-Organometallic Catalyst based on SBA-15 (Pd-TCH@SBA-15) and their Application in the preparation of Organic compounds.**

**Applications of SBA-15 supported Pd metal catalysts as nanoreactors in C–C coupling reactions**

## Synthesis of Pd/SBA-15 catalyst employing surface-bonded vinyl as a reductant and its application in the hydrogenation of nitroarenes

**Pd@SBA-15 nanocomposite catalyst: Synthesis and efficient solvent-free semihydrogenation of phenylacetylene under mild conditions**

**Preparation of Pd-Diimine@SBA-15 and Its Catalytic Performance for the Suzuki Coupling Reaction**

Yu, J.; Shen, A.; Cao, Y.; Lu, G. Preparation of Pd-Diimine@SBA-15 and Its Catalytic Performance for the Suzuki Coupling Reaction. *Catalysts* **2016**, *6*, 181.

**Palladium-doped mesoporous silica SBA-15 modified in carbon-paste electrode as a sensitive voltammetric sensor for detection of oxalic acid**

**Preparation and characterization of SBA-15 supported Pd catalyst for CO oxidation**

# **Green preparation of Pd nanoparticles on SBA-15 via supercritical fluid deposition and application on Suzuki–Miyaura cross-coupling reaction**

# **Fabrication of Palladium Nanoparticles/Graphene/SBA-15 Nanocomposites for the Catalysis of Suzuki Coupling Reaction**

# **Pd immobilized on amidoxime-functionalized Mesoporous SBA-15: A novel and highly active heterogeneous catalyst for Suzuki–Miyaura coupling reactions**
