# Supplementary material for: Pd Doped on TCH@SBA-15 Nanocomposites: Fabrication and Application as a New Organometallic Catalyst in the Three-Component Synthesis of N-Benzo-imidazo- or -thiazole-1,3-thiazolidinones
Source: Front Chem. 2021 Oct 4;9:723207. doi: 10.3389/fchem.2021.723207 (PMC8524445; doi:10.3389/fchem.2021.723207)
Supplement: Supplementary file 2 [file DataSheet1.docx]

**Pd doped on nanocomposite TCH@SBA-15: fabrication and application as a new organometallic catalyst in three-component synthesis of *N*-benzo- imidazo- or -thiazole-1,3-thiazolidinones**

Mehdi Kalhor*, Akbar Dadras

*Department of Chemistry, Payame Noor* *University, Tehran, 19395-4697, Iran.*

* Corresponding author. Tel.: +98 2537179170; fax: +98 2537179170; e-mail: [mekalhor@gmail.com](mailto:mekalhor@gmail.com)

**Supporting Information**

^1^H, ^13^C NMR and Mass Spectra of **4a-n**

**3-(1*H*-benzo[*d*]imidazol-2-yl)-2-phenylthiazolidin-4-one (4a)**

IR (KBr) (*ν*_max_): 3360 (NH), 1700 (C=O), 1532 (C=N), 1381, 1269 (C=C), 1117 (C-N), 655 (C-S-C) cm^−1^; ^1^H-NMR (300 MHz, DMSO-*d_6_*) *δ_H_*: 3.93 (1H, d, *J* = 16.53 Hz, SCH_2_), 4.17 (1H, d, *J* = 16.56 Hz, SCH_2_), 6.77 (1H, s, CH), 7.06-7.11 (2H, m, H-Ar), 7.24-7.39 (6H, m, H-Ar), 7.50 (1H, d, *J* = 7.50 Hz, H-Ar), 12.44 (1H, s, NH) ppm; ^13^C-NMR (75 MHz, DMSO-*d_6_*) *δ*_C_: 32.0, 61.5, 111.2, 121.6, 125.3, 127.9, 128.6, 134.4, 141.4, 144.4, 171.7 ppm; MS (m/z, %): 295.1 (M^+^, 40), 249.1 (31), 220.1 (44), 133.1 (100), 105.1 (69), 77.1 (40).

**3-(1*H*-benzo[*d*]imidazol-2-yl)-2-(4-nitrophenyl)thiazolidin-4-one (4b)**

IR (KBr) (*ν*_max_): 3477 (NH), 1708 (C=O), 1617, 1514 (C=N), 1520, 1349 (NO_2_), 1451, 1345, 1270 (C=C), 1114 (C-N), 626 (C-S-C) cm^−1^; ^1^H-NMR (300 MHz, DMSO-*d_6_*) *δ*_H_: 3.96 (1H, d, *J* = 16.53 Hz, SCH_2_), 4.20 (1H, d, *J* =16.53 Hz, SCH_2_), 6.90 (1H, s, CH), 7.03-7.13 (2H, m, H-Ar), 7.33 (1H, d, *J* = 7.68 Hz, H-Ar), 7.50 (1H, d, *J* = 7.53 Hz, H-Ar), 7.66 (2H, d, *J* = 8.70 Hz, H-Ar), 8.16 (2H, d, *J* = 8.67 Hz, H-Ar) 12.47 (1H, s, NH) ppm; ^13^C-NMR (75 MHz, DMSO-*d*_6_) *δ*_C_: 32.0, 60.7, 111.2, 121.7, 122.4, 123.2, 124.0, 126.6, 130.3, 144.3, 146.9, 149.0, 152.9, 171.5 ppm; MS (m/z, %): 340.2 (M^+^, 100), 294.1 (92), 219.1 (50), 164.1 (26), 118.1 (41), 91.1 (27), 77.1 (13).

**3-(1*H*-benzo[d]imidazol-2-yl)-2-(3-nitrophenyl)thiazolidin-4-one (4c)**

IR (KBr) (*ν*_max_): 3336 (NH), 1704 (C=O), 1620 (C=N), 1520, 1349 (NO_2_), 1262 (C=C), 1119 (C-N), 617 (C-S-C) cm^−1^;^1^H-NMR (300 MHz, DMSO-*d_6_*) *δ_H_*: 3.94 (1H, d, *J* = 16.56 Hz, SCH_2_), 4.24 (1H, d, *J* =16.53 Hz, SCH_2_), 6.93 (1H, s, CH), 7.03-7.13 (2H, m, H-Ar), 7.34 (1H, d, *J* = 7.50 Hz, H-Ar), 7.50 (1H, d, *J* = 7.35 Hz, H-Ar), 7.62 (1H, t, *J* = 7.98 Hz, H-Ar ), 7.85 (1H, d, *J* = 7.92 Hz, H-Ar), 8.10 (1H, d br, H-Ar), 8.29 (1H, t br, H-Ar ), 12.47 (1H, s, NH) ppm; ^13^C-NMR (75 MHz, DMSO-*d*_6_) *δ*_C_: 32.0, 60.6, 111.9, 117.6, 120.5, 121.6, 121.8, 122.9, 130.3, 131.8, 132.7, 139.8, 143.9, 144.3, 147.9, 171.6 ppm; MS (m/z, %): 340 (M^+^, 100), 294.1 (86), 265.1 (39), 219.1 (37), 164.1 (27), 118 (43), 91 (28).

**3-(1*H*-benzo[d]imidazol-2-yl)-2-(2-nitrophenyl)thiazolidin-4-one (4d)**

IR (KBr) (*ν*_max_): 3437, 3338 (NH), 2929 (C-H), 1703 (C=O), 1650, 1540 (C=N), 1520, 1338 (NO_2_), 1455, 1369 (C=C), 1118 (C-N), 1024, 668 (C-S-C) cm^−1^; ^1^H-NMR (500 MHz, DMSO-*d_6_*) *δ_H_*: 3.39 (1H, d, *J* = 16.55 Hz, SCH_2_), 4.21 (1H, d, *J* = 16.55 Hz, SCH_2_), 7.06 (3H, d br, CH and H-Ar), 7.43 (2H, s br, H-Ar), 7.47 (1H, d, *J* = 7.90 Hz, H-Ar), 7.55 (1H, t, *J* = 7.55 Hz, H-Ar), 8.17 (1H, d, *J* = 8.10 Hz, H-Ar), 12.51 (1H, br, NH) ppm; ^13^C-NMR (125 MHz, DMSO-*d_6_*) *δ*_C_: 32.2, 58.2, 122.2, 126.0, 126.1, 129.8 (2C), 135.5, 137.2 (2C), 144.8, 146.8, 172.1 ppm; MS (m/z, %): 340 (M^+^, 30), 294 (32), 220 (65), 206 (30), 178 (25), 160 (100), 133 (25), 104 (18), 90 (20), 77 (18).

**3-(1*H*-benzo[*d*]imidazol-2-yl)-2-(4-bromophenyl)thiazolidin-4-one (4e)**

IR (KBr) (*ν*_max_): 3413 (NH), 1706 (C=O), 1618, 1535 (C=N), 1486, 1450, 1369, 1270 (C=C), 1227, 1117 (C-N), 1006, 738, 722, 659 (C-S-C), 497 cm^−1^; ^1^H-NMR (300 MHz, DMSO-*d*_6_) *δ*_H_: 3.92 (1H, d, *J* = 16.53 Hz, SCH_2_), 4.18 (1H, d, *J* =16.53 Hz, SCH_2_), 6.74 (1H, s, CH), 7.04-7.13 (2H, m, H-Ar), 7.34 (3H, d, *J* = 8.37 Hz, H-Ar), 7.49 (3H, d, *J* = 8.40 Hz, H-Ar), 12.42 (1H, s, NH) ppm; ^13^C-NMR (75 MHz, DMSO-*d*_6_) *δ*_C_: 32.0, 61.0, 112.0, 119.8, 121.0, 121.6, 127.6, 128.9, 131.2, 131.5, 141.0, 144.3, 171.6 ppm; MS (m/z, %): 375.1 (M^+^, 25), 327.1 (23), 300.1 (100), 144.1 (10), 118.1 (35), 91.1 (28).

**3-(1*H*-benzo[d]imidazol-2-yl)-2-(2-chlorophenyl)thiazolidin-4-one (4f)**

IR (KBr) (*ν*_max_): 3444, 3349 (NH), 2925 (C-H), 1683 (C=O), 1535 (C=N), 1447, 1303, 1269 (C=C), 1170 (C-N), 743, 647 (C-S-C) cm^−1^; ^1^H-NMR (500 MHz, DMSO-*d_6_*) *δ_H_*: 3.97 (1H, d, *J* = 16.50 Hz, SCH_2_), 4.11 (1H, d, *J* = 16.45 Hz, SCH_2_), 6.85 (1H, s, CH), 7.06-7.15 (3H, m, H-Ar), 7.24 (1H, t, *J* = 7.55 Hz, H-Ar), 7.30 (1H, t, *J* = 7.60 Hz, H-Ar), 7.38 (1H, d, *J* = 7.90 Hz, H-Ar), 7.53 (2H, t, *J* = 7.60 Hz, H-Ar), 12.52 (1H, s, NH) ppm; ^13^C-NMR (125 MHz, DMSO-*d*_6_) *δ*_C_: 32.1, 59.5, 112.4, 118.2, 122.0, 122.2, 125.2, 128.1, 129.9, 130.5, 131.5, 133.3, 138.3, 140.2, 144.7, 172.2 ppm; MS (m/z, %): 329 (M^+^, 10), 294 (100), 252 (30), 220 (94), 135 (30), 118 (20), 91 (18).

**3-(1*H*-benzo[*d*]imidazol-2-yl)-2-(4-methoxyphenyl) thiazolidin-4-one (4g)**

IR (KBr) (*ν*_max_): 3478 (NH), 1707 (C=O), 1638, 1617, 1540, 1511 (C=N), 1452, 1373 (C=C), 1270, 1254 (C-O), 1178 (C-N), 1116, 1026, 843, 726, 665 (C-S-C), 607 cm^−1^; ^1^H-NMR (300 MHz, DMSO-*d_6_*) *δ_H_*: 3.68 ( 3H, s, OCH_3_), 3.91 (1H, d, *J* = 16.53 Hz, SCH_2_), 4.17 (1H, d, *J* =16.53 Hz, SCH_2_), 6.71 (1H, s, CH), 6.83 (2H, d*, J* = 8.61 Hz, H-Ar), 7.04-7.12 (2H, m, H-Ar), 7.31 (2H, d, *J* = 8.61 Hz, H-Ar), 7.37 (1H, d, *J* = 7.53 Hz, H-Ar), 7.48 ( 1H, d, *J* = 7.14 Hz, H-Ar ), 12.41 (1H, s, NH) ppm; ^13^C-NMR (75 MHz, DMSO-*d*_6_) *δ*_C_: 32.2, 55.0, 61.5, 112.0, 113.7, 113.9, 121.6, 126.9, 133.2, 144.4, 158.9, 171.6 ppm; MS (m/z, %): 325.2 (M^+^, 100), 279.1 (59), 250.1 (72), 165.1 (55), 135.1 (51), 118.1 (33), 91 (22).

**3-(1*H*-benzo[*d*]imidazol-2-yl)-2-(4-methylphenyl) thiazolidin-4-one (4h)**

IR (KBr) (*ν*_max_): 3413 (NH), 1707 (C=O), 1638, 1617 (C=N), 1531, 1511, 1452, 1373, 1270 (C=C), 1254, 1178 (C-N), 726, 665, 607 (C-S-C) cm^−1^; ^1^H-NMR (300 MHz, DMSO-*d_6_*) *δ_H_*: 2.21 (3H, s, CH_3_), 3.91 (1H, d, *J* = 16.56 Hz, SCH_2_), 4.15 (1H, d, *J* =16.53 Hz, SCH_2_), 6.71 (1H, s, CH), 7.03-7.11 (4H, m, CH), 7.27 (2H, d, *J* = 7.89 Hz, H-Ar), 7.38 (1H, d, *J* = 7.17 Hz, H-Ar), 7.51 (1H, d, *J* = 7.62 Hz, H-Ar, 12.41 (1H, br, NH),) ppm; ^13^C-NMR (75 MHz, DMSO-*d*_6_) *δ*_C_: 20.6, 32.12, 61.5, 119.8, 121.6, 125.3, 126.6, 128.9, 129.1, 137.0, 138.4, 171.6 ppm; MS (m/z, %): 309.2 (M^+^, 100), 263.1 (71), 234.1 (75), 175.1 (27), 135.1 (74), 91 (26).

**3-(1*H*-benzo[*d*]imidazol-2-yl)-2-(3,4-dimethoxyphenyl) thiazolidin-4-one (4i)**

IR (KBr) (*ν*_max_): 3400 (NH), 1704 (C=O), 1637, 1617, 1537 (C=N), 1514, 1452 (C=C), 1273, 1256, 1239 (C-O), 1141 (C-N), 648 (C-S-C), 616, 478 cm^−1^; ^1^H-NMR (300 MHz, DMSO-*d_6_*) *δ_H_*: 3.67 ( 3H, s, OCH_3_ ), 3.72 ( 3H, s, OCH_3_), 3.90 (1H, d, *J* = 16.50 Hz, SCH_2_), 4.16 (1H, d, *J* =16.53 Hz, SCH_2_), 6.70 (1H, s, CH), 6.81 (2H, s*,* H-Ar), 7.05-7.13 (3H, m, H-Ar), 7.38 (1H, d, *J* = 7.47 Hz, H-Ar), 7.48 (1H, d, *J* = 7.29 Hz, H-Ar), 12.42 (1H, s, NH) ppm; ^13^C-NMR (75 MHz, DMSO-*d*_6_) *δ*_C_: 32.1, 55.4 (2C), 61.6, 109.7, 111.4, 111.8, 117.0, 117.7, 121.5, 121.7, 132.7, 133.4, 140.0, 144.4, 148.5, 148.8, 171.7 ppm; MS (m/z, %): 355.2 (M^+^, 100), 313.2 (61), 280.2 (50), 192.1 (17), 165.1 (81), 118.1 (30), 91.1 (18).

**3-(Benzo[*d*]thiazol-2-yl)-2-(4-nitrophenyl)thiazolidin-4-one (4j)**

IR (KBr) (*ν*_max_): 1690 (C=O), 1617, 1537 (C=N), 1467, 1369, 1274 (C=C), 1349, 1530 (NO_2_), 1228 (C-N), 658 (C-S-C) cm^−1^; ^1^H-NMR (300 MHz, DMSO-*d_6_*) *δ*_H_: 4.03 (1H, d, *J* = 16.73 Hz, SCH_2_), 4.28 (1H, d, *J* = 16.67 Hz, SCH_2_), 7.03 (1H, s, CH), 7.29-7.41 (2H, m, H-Ar), 7.61 (1H, d, *J* = 7.53 Hz, H-Ar), 7.68 (2H, d, *J* = 8.76 Hz, H-Ar), 8.01 (1H, q, *J* = 1.18 Hz, H-Ar), 8.16 (2H, d, *J* = 8.76 Hz, H-Ar) ppm; ^13^C-NMR (75 MHz, DMSO-*d*_6_) *δ*_C_: 171.6, 156.0, 148.5, 147.5, 147.0, 131.2, 126.7, 126.4, 124.5, 124.0, 122.0, 121.2, 61.7, 31.9 ppm; MS (m/z, %): 357.1 (M^+^, 3.4), 108 (22.9), 69 (23.6), 46 (100).

**3-(Benzo[*d*]thiazol-2-yl)-2-(3-nitrophenyl)thiazolidin-4-one (4k)**

IR (KBr) (*ν*_max_): 1690 (C=O), 1617, 1537 (C=N), 1467, 1369, 1274 (C=C), 1349, 1530 (NO_2_), 1228 (C-N), 658 (C-S-C) cm^−1^; ^1^H-NMR (300 MHz, DMSO-*d_6_*) *δ*_H_: 4.02 (1H, d, *J* = 16.73 Hz, SCH_2_), 4.32 (1H, d, *J* = 16.71 Hz, SCH_2_), 7.06 (1H, s, CH), 7.29-7.40 (2H, m, H-Ar), 7.62 (2H, t, *J* = 7.78 Hz, H-Ar), 7.86 (1H, d, *J* = 7.77 Hz, H-Ar), 8.00 (1H, d, *J* = 7.45 Hz, H-Ar), 8.10 (1H, q, *J* = 1.35 Hz, H-Ar), 8.34 (1H, s, H-Ar) ppm; ^13^C-NMR (75 MHz, DMSO-*d*_6_) *δ*_C_: 171.6, 155.9, 147.8, 147.5, 143.4, 131.8, 131.2, 130.3, 126.4, 124.4, 122.9, 121.9, 121.2, 120.8, 61.8, 31.8 ppm; MS (m/z, %): 357.6 (M^+^, 100), 315.6 (57), 284.6 (62.5), 255.6 (32.6), 237.6 (32.7), 181.5 (33.2), 135 (56).

**3-(Benzo[*d*]thiazol-2-yl)-2-(4-methylphenyl)thiazolidin-4-one (4l)**

IR (KBr) (*ν*_max_): 1690 (C=O), 1617, 1537 (C=N), 1467, 1369, 1274 (C=C), 1228 (C-N), 658 (C-S-C) cm^−1^; ^1^H-NMR (300 MHz, DMSO-*d_6_*) *δ*_H_: 2.23 (3H, s, Me), 3.98 (1H, d, *J* = 16.78 Hz, SCH_2_), 4.23 (1H, d, *J* = 16.75 Hz, SCH_2_), 6.86 (1H, s, CH), 7.10 (2H, d, *J* = 7.99 Hz, H-Ar), 7.25-7.42 (4H, m, H-Ar), 7.64 (1H, d, *J* = 7.81 Hz, H-Ar), 7.99 (1H, q, *J* = 0.68 Hz, H-Ar) ppm; ^13^C-NMR (75 MHz, DMSO-*d*_6_) *δ*_C_: 171.7, 155.9, 147.6, 138.1, 137.3, 131.2, 129.2, 126.3, 125.3, 124.3, 121.9, 121.2, 62.6, 31.8, 20.6 ppm; MS (m/z, %): 326.5 (M^+^, 93.2), 284.5 (50.5), 251.5 (85), 181.5 (32.1), 135.5 (100), 91.5 (24.3), 69.4 (25.8).

**3-(Benzo[*d*]thiazol-2-yl)-2-(4-bromophenyl)thiazolidin-4-one (4m)**

IR (KBr) (*ν*_max_): 1690 (C=O), 1617, 1537 (C=N), 1467, 1369, 1274 (C=C), 1228 (C-N), 658 (C-S-C) cm^−1^; ^1^H-NMR (300 MHz, DMSO-*d_6_*) *δ*_H_: 3.99 (1H, d, *J* = 16.76 Hz, SCH_2_), 4.26 (1H, d, *J* = 16.73 Hz, SCH_2_), 6.89 (1H, s, CH), 7.30-7.41 (4H, m, H-Ar), 7.50 (2H, d, *J* = 8.41 Hz, H-Ar), 7.63 (1H, d, *J* = 8.75 Hz, H-Ar), 8.00 (1H, d, *J* = 7.74 Hz, H-Ar) ppm; ^13^C-NMR (75 MHz, DMSO-*d*_6_) *δ*_C_: 171.6, 155.9, 147.5, 140.6, 131.5, 131.2, 127.7, 126.3, 124.4, 121.9, 121.2, 121.0, 62.1, 31.8 ppm; MS (m/z, %): 392.4 (M^+^, 58.4), 350.4 (42.5), 317.4 (83.2), 181.5 (49.9), 161.5 (22.4), 135.5 (100), 108.4 (32.9).

**3-(Benzo[*d*]thiazol-2-yl)-2-(4-methoxyphenyl)thiazolidin-4-one (4n)**

IR (KBr) (*ν*_max_): 1690 (C=O), 1617, 1537 (C=N), 1467, 1369, 1274 (C=C), 1228 (C-N), 658 (C-S-C) cm^−1^; ^1^H-NMR (300 MHz, DMSO-*d_6_*) *δ*_H_: : 3.69 (3H, s, OMe), 3.98 (1H, d, *J* = 16.81 Hz, SCH_2_), 4.26 (1H, d, *J* = 16.79 Hz, SCH_2_), 6.85 (1H, s, CH), 6.85 (2H, d, *J* = 7.51 Hz, H-Ar), 7.29-7.42 (4H, m, H-Ar), 7.65 (1H, d, *J* = 7.97 Hz, H-Ar), 7.99 (1H, d, *J* = 7.69 Hz, H-Ar) ppm; ^13^C-NMR (75 MHz, DMSO-*d*_6_) *δ*_C_: 171.6, 158.9, 155.9, 147.6, 132.9, 131.2, 127.0, 126.3, 124.3, 121.8, 121.2, 113.9, 62.5, 55.0, 31.9 ppm; MS (m/z, %): 342.6 (M^+^, 99.9), 300.6 (71.6), 267.6 (80.5), 208.5 (68.8), 181.5 (59.6), 151.5 (54.7), 135.5 (100), 108.4 (32.8).

**Fig. S1. The ^1^H NMR spectrum of 3-(1*H*-benzo[*d*]imidazol-2-yl)-2-phenylthiazolidin-4-one (4a)**


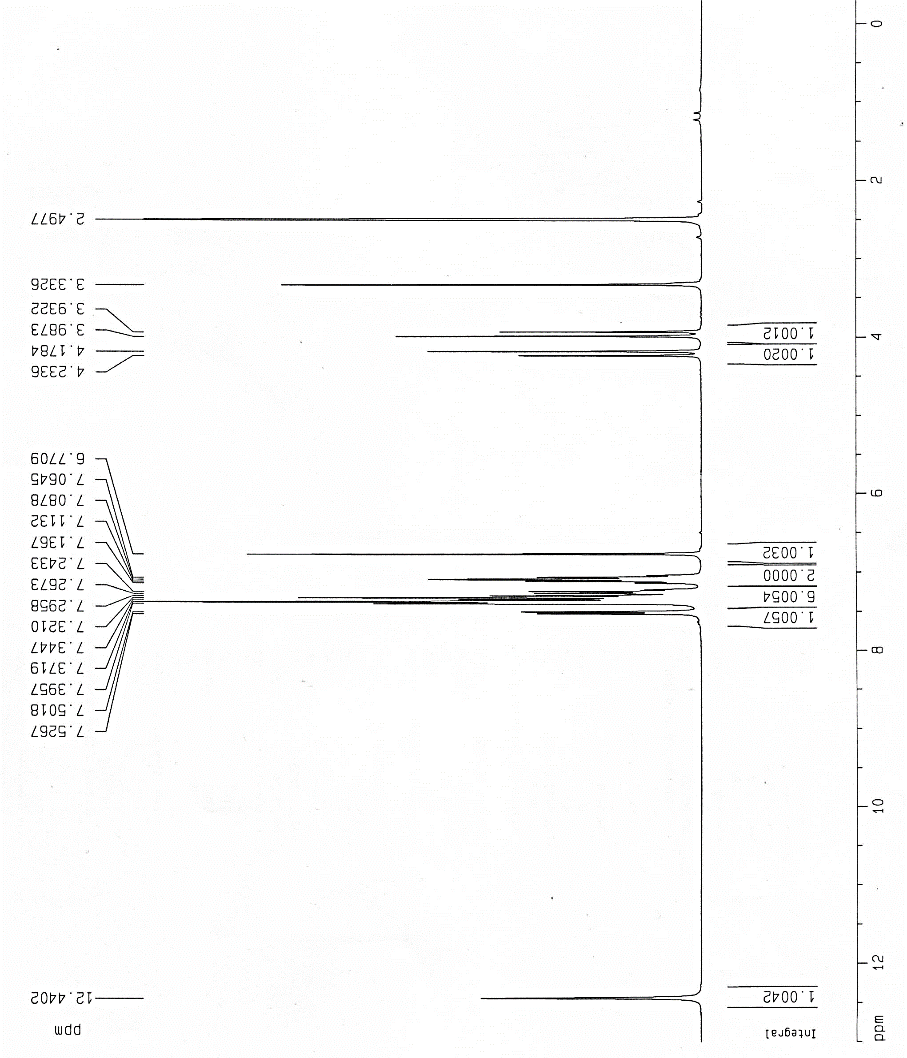


**Fig. S2. The ^13^C NMR spectrum of 3-(1*H*-benzo[*d*]imidazol-2-yl)-2-phenylthiazolidin-4-one (4a)**


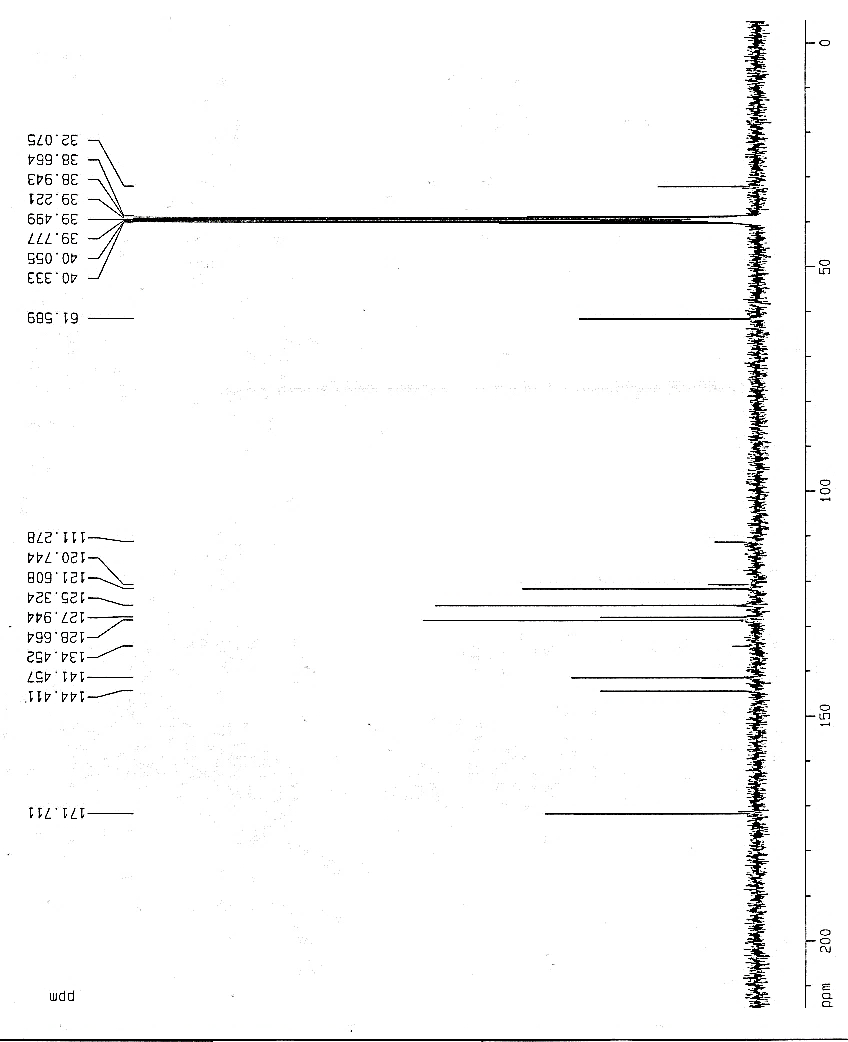


**Fig. S3. The Mass spectrum of 3-(1*H*-benzo[*d*]imidazol-2-yl)-2-phenylthiazolidin-4-one (4a)**

**Fig. S4. The ^1^H NMR spectrum of 3-(1*H*-benzo[*d*]imidazol-2-yl)-2-(4-nitrophenyl)thiazolidin-4-one (4b)**

**
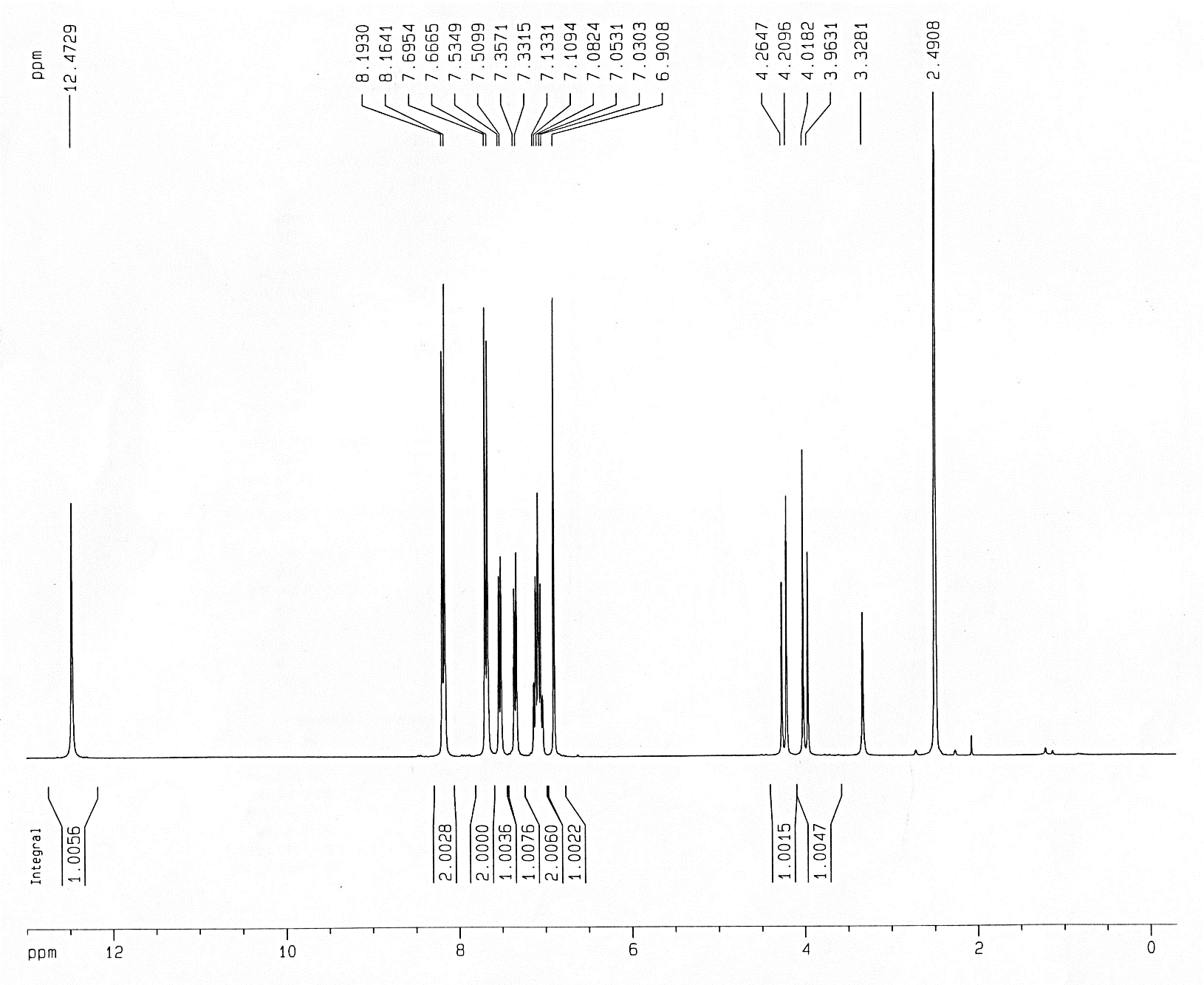
**

**Fig. S5. The ^13^C NMR spectrum of 3-(1*H*-benzo[*d*]imidazol-2-yl)-2-(4-nitrophenyl)thiazolidin-4-one (4b)**

**
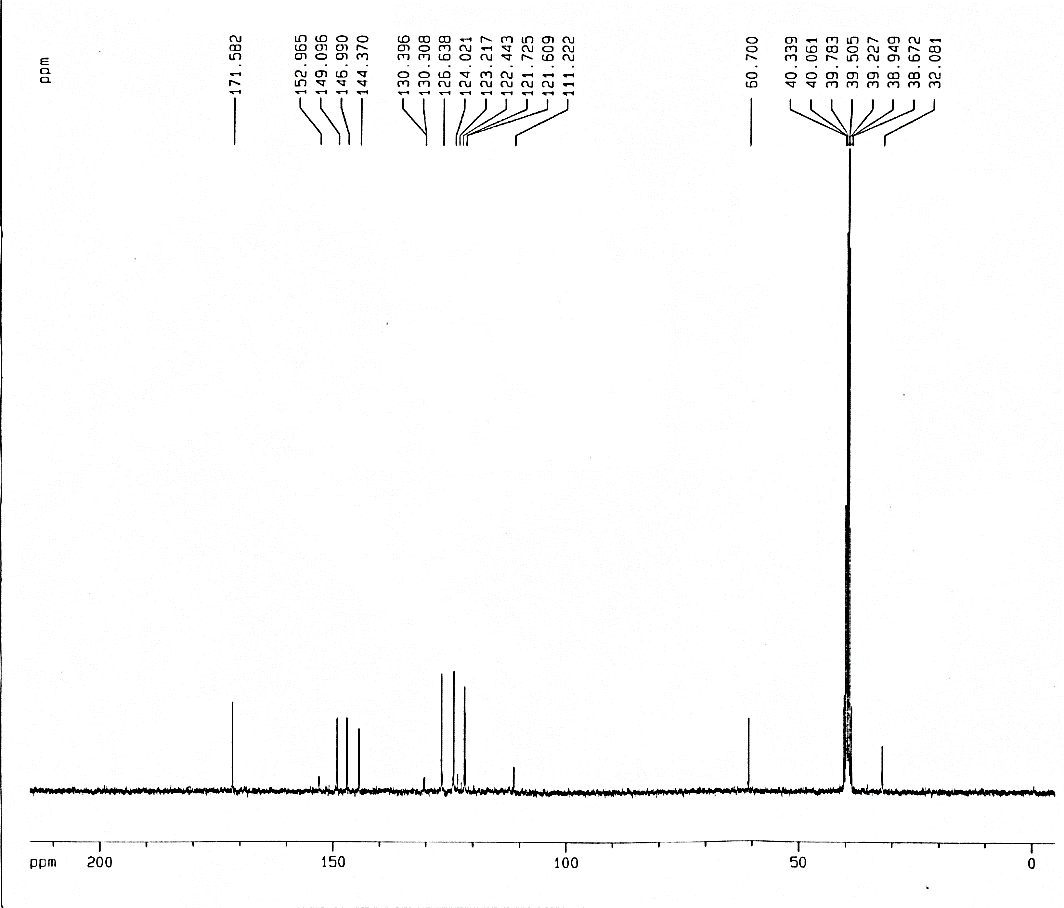
**

**Fig. S6. The Mass spectrum of 3-(1*H*-benzo[*d*]imidazol-2-yl)-2-(4-nitrophenyl)thiazolidin-4-one (4b)**

**Fig. S7. The ^1^H NMR spectrum of 3-(1*H*-benzo[*d*]imidazol-2-yl)-2-(3-nitrophenyl)thiazolidin-4-one (4c)**

**
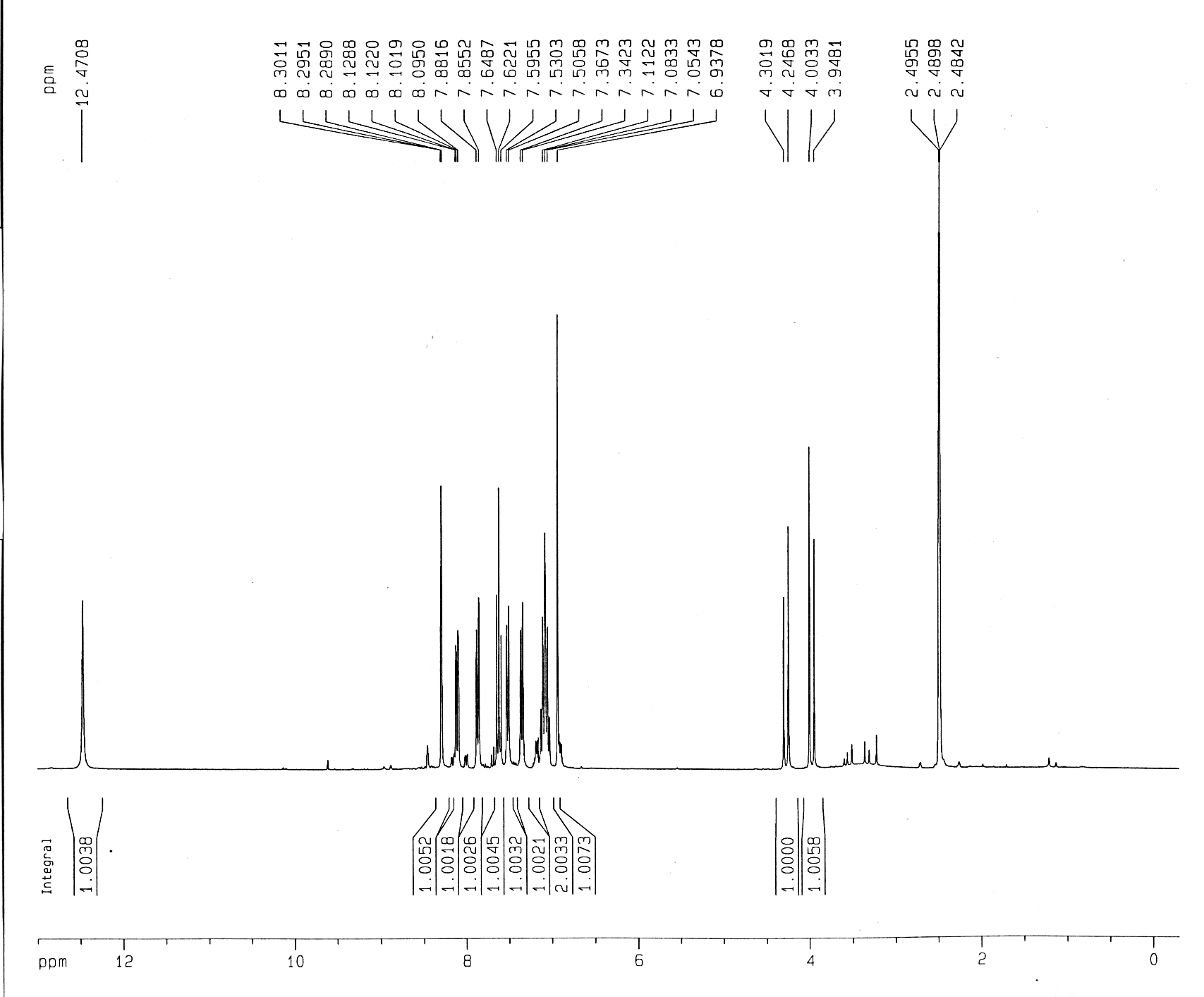
**

**Fig. S8. The ^13^C NMR spectrum of 3-(1*H*-benzo[*d*]imidazol-2-yl)-2-(3-nitrophenyl)thiazolidin-4-one (4c)**


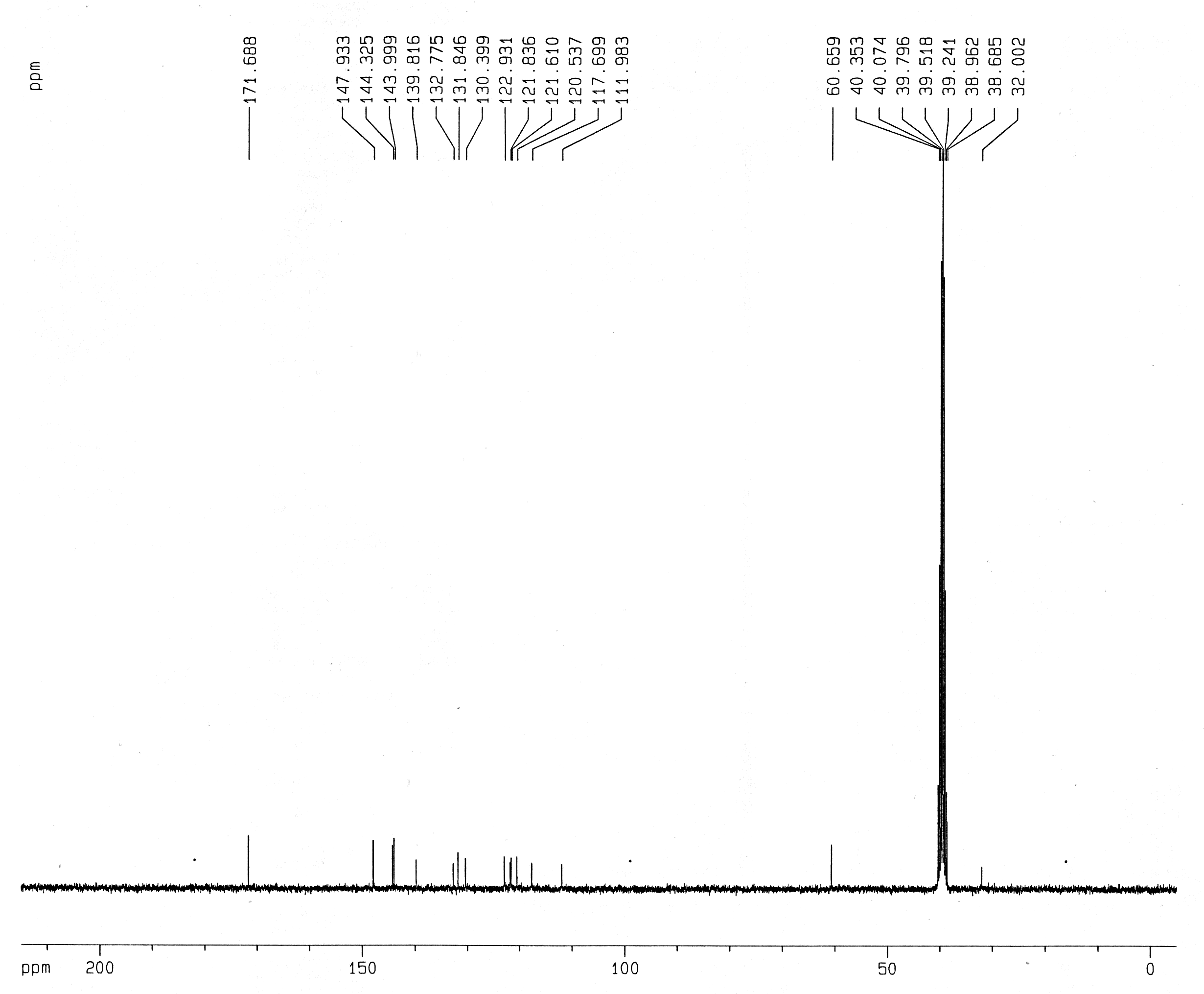


**Fig. S9. The Mass spectrum of 3-(1*H*-benzo[*d*]imidazol-2-yl)-2-(3-nitrophenyl)thiazolidin-4-one (4c)**

**Fig. S10. The ^1^H NMR spectrum of 3-(1*H*-benzo[*d*]imidazol-2-yl)-2-(2-nitrophenyl)thiazolidin-4-one (4d)**

**
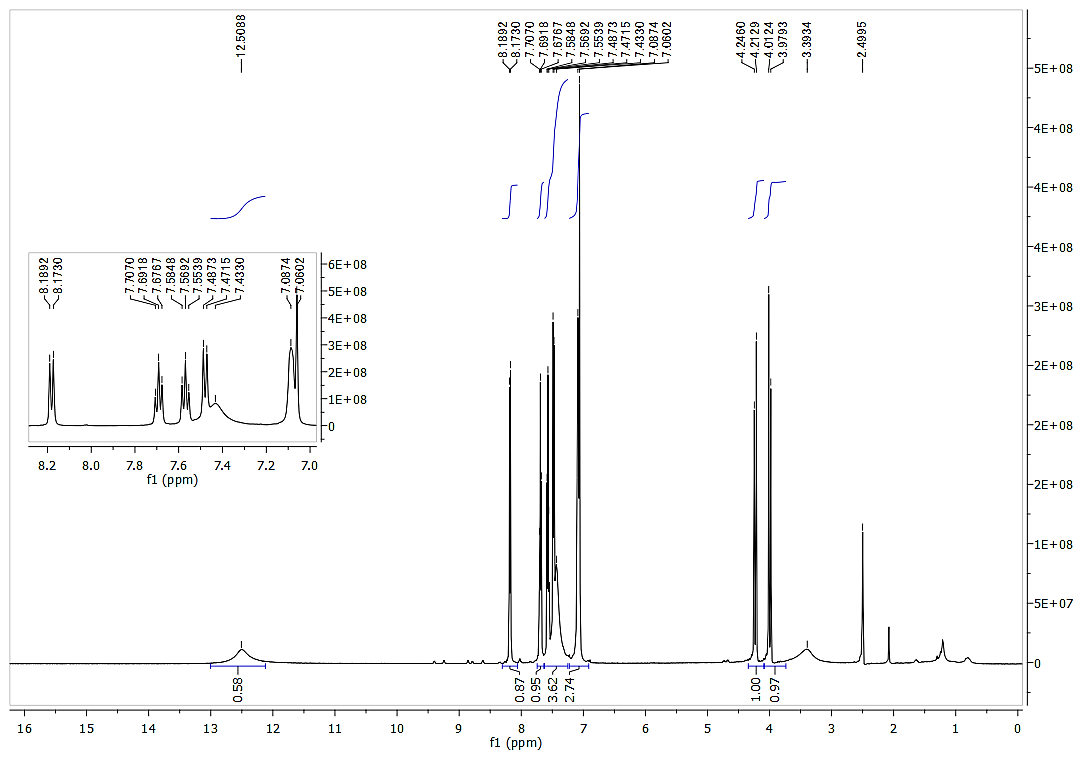
**

**Fig. S11. The ^13^C NMR spectrum of 3-(1*H*-benzo[*d*]imidazol-2-yl)-2-(2-nitrophenyl)thiazolidin-4-one (4d)**

**
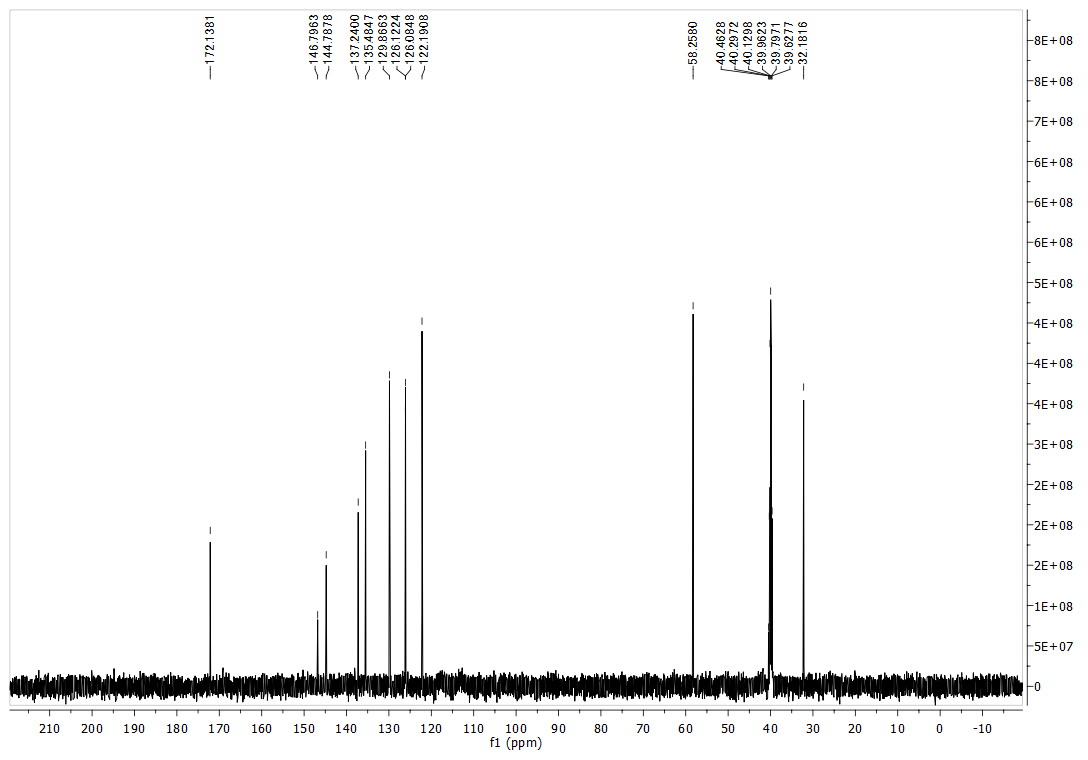
**

**Fig. S12. The Mass spectrum of 3-(1*H*-benzo[*d*]imidazol-2-yl)-2-(2-nitrophenyl)thiazolidin-4-one (4d)**

**
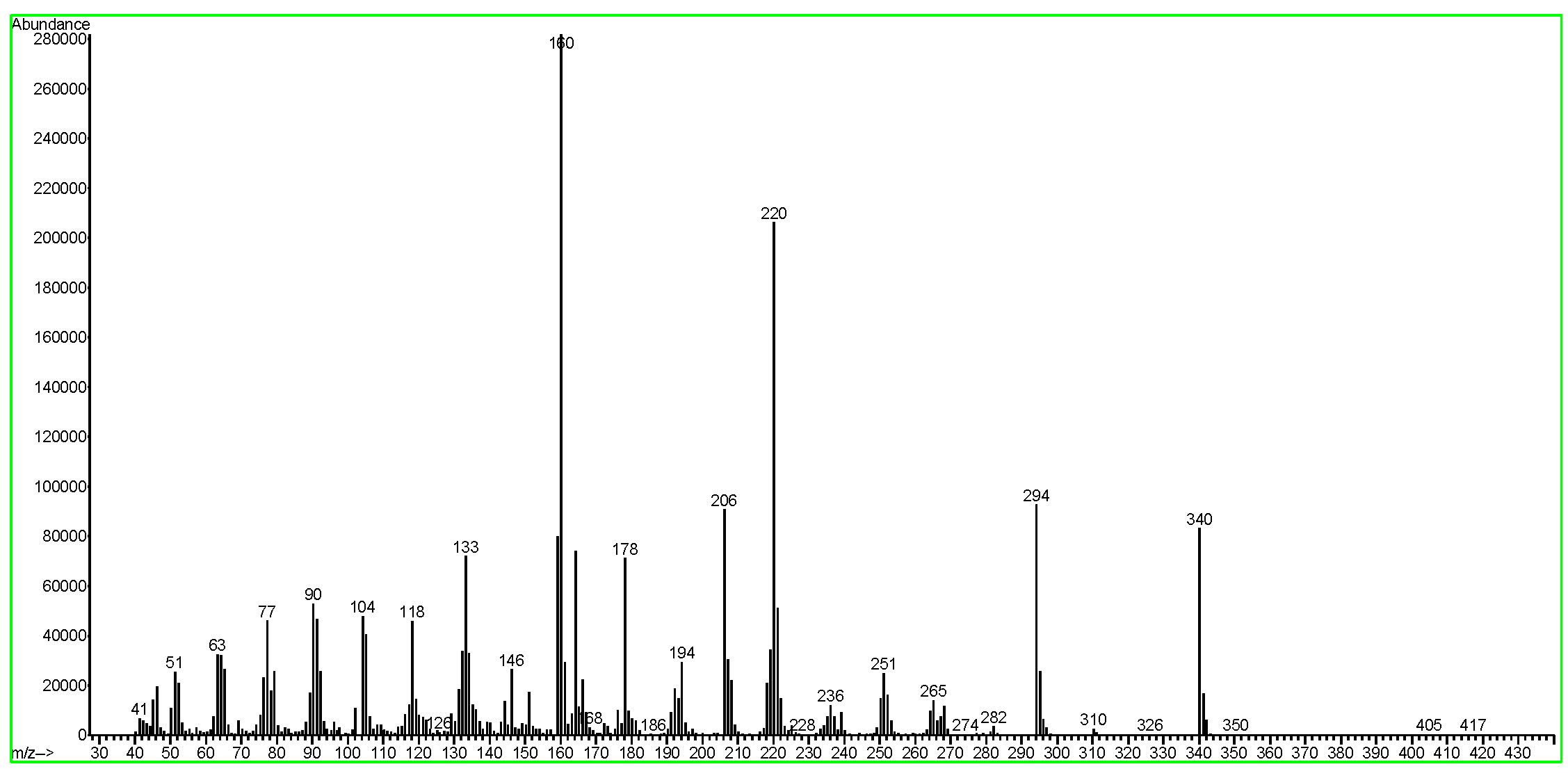
**

**Fig. S13. The ^1^H NMR spectrum of 3-(1*H*-benzo[*d*]imidazol-2-yl)-2-(4-bromophenyl)thiazolidin-4-one (4e)**

**
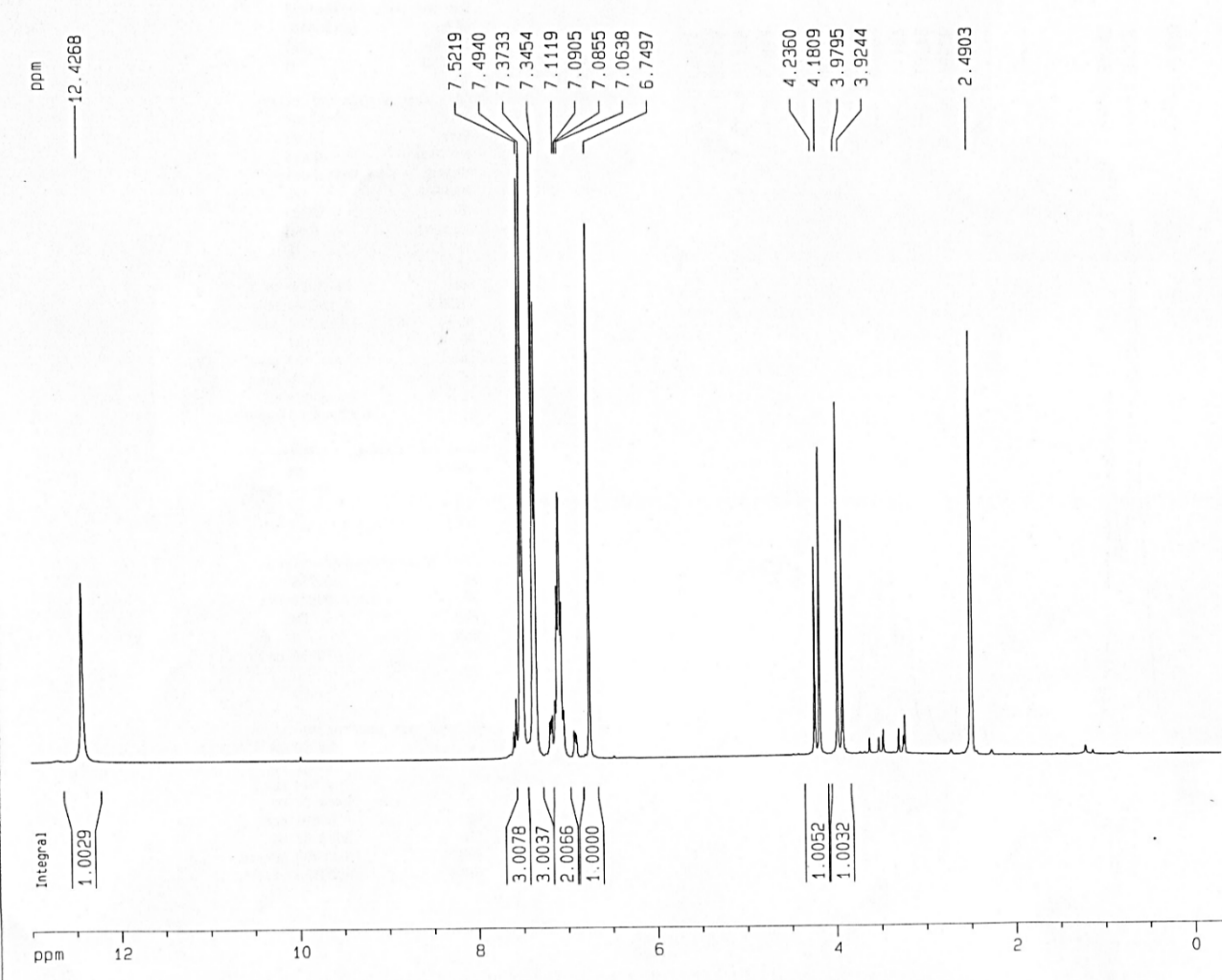
**

**Fig. S14. The ^13^C NMR spectrum of 3-(1*H*-benzo[*d*]imidazol-2-yl)-2-(4-bromophenyl)thiazolidin-4-one (4e)**


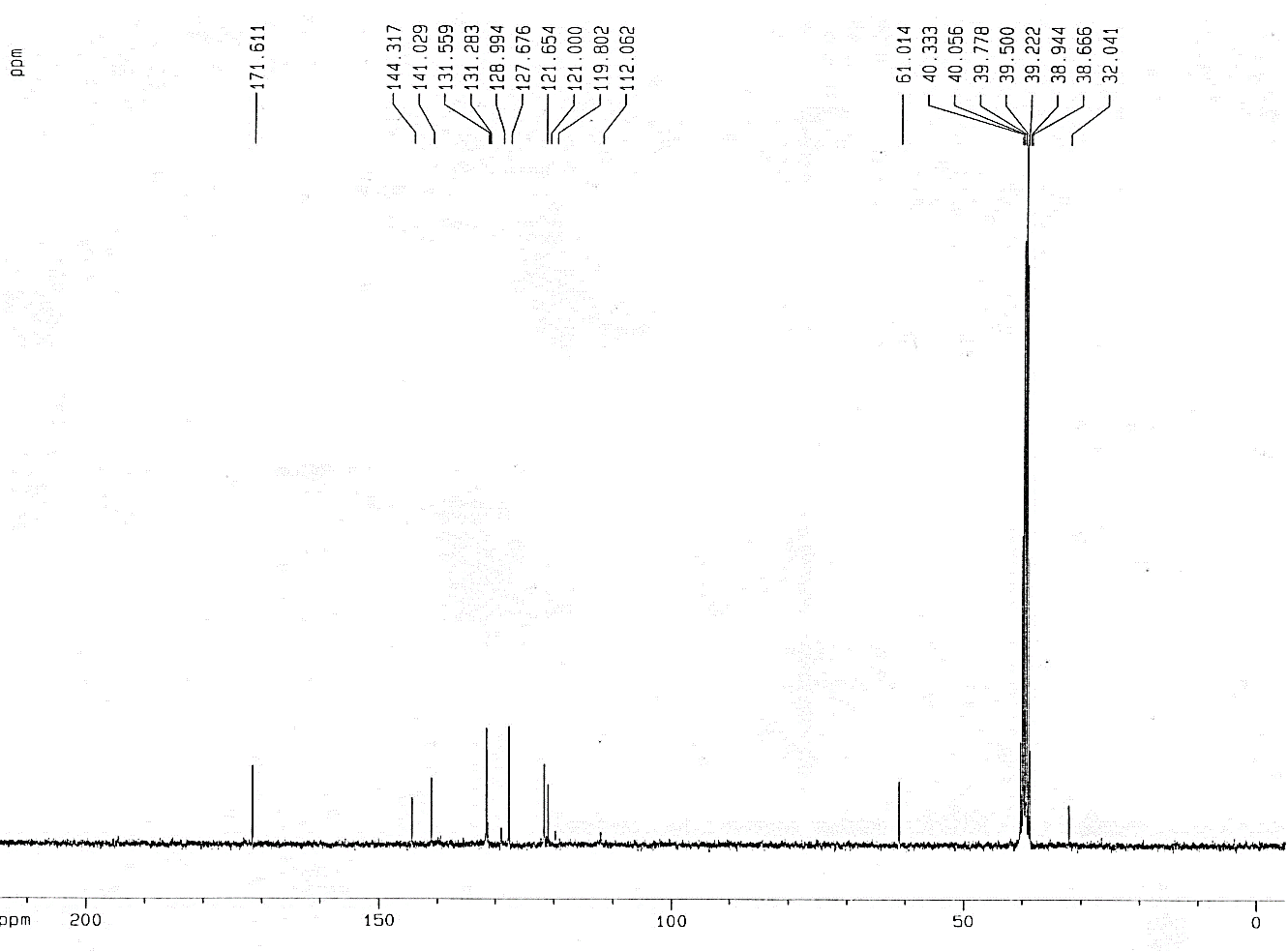


**Fig. S15. The Mass spectrum of 3-(1*H*-benzo[*d*]imidazol-2-yl)-2-(4-bromophenyl)thiazolidin-4-one (4e)**

**Fig. S16. The ^1^H NMR spectrum of 3-(1*H*-benzo[*d*]imidazol-2-yl)-2-(2-chlorophenyl)thiazolidin-4-one (4f)**


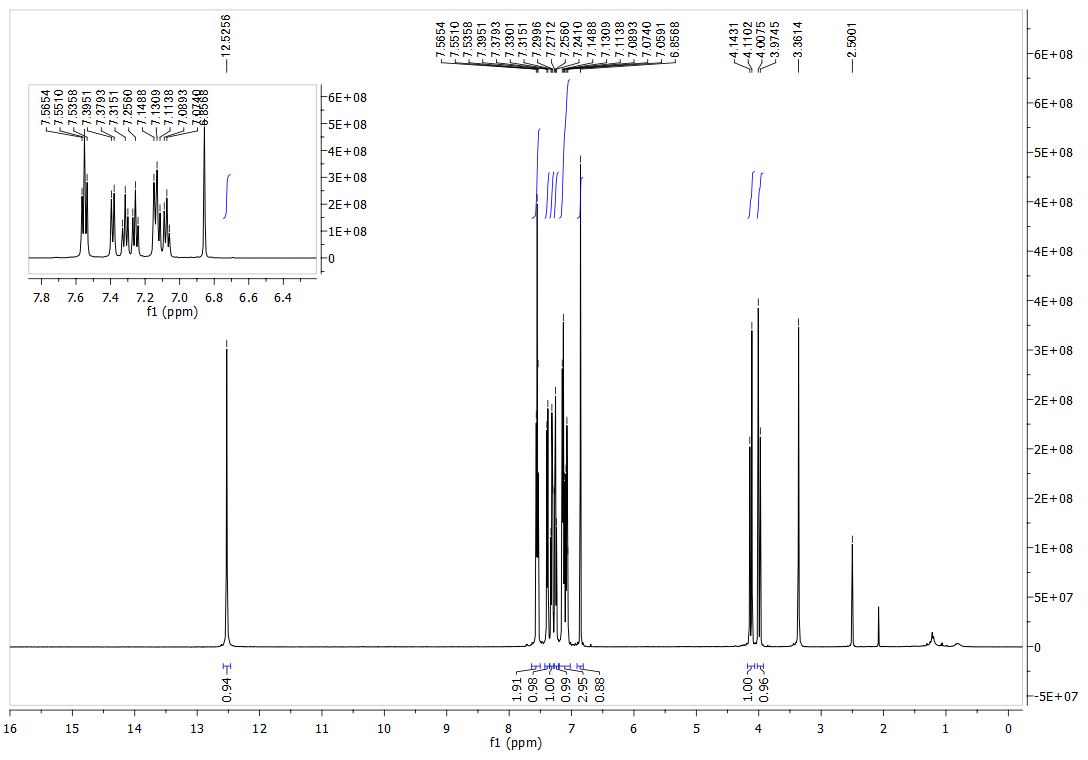


**Fig. S17. The ^13^C NMR spectrum of 3-(1*H*-benzo[*d*]imidazol-2-yl)-2-(2-chlorophenyl)thiazolidin-4-one (4f)**


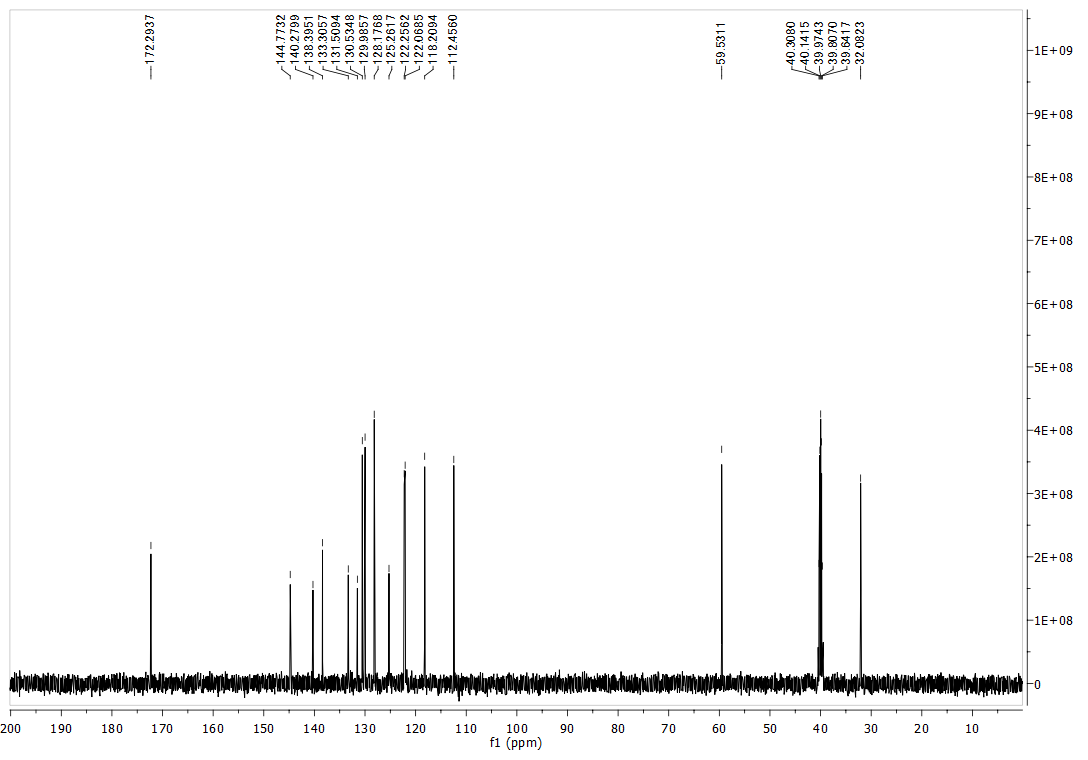


**Fig. S18. The Mass spectrum of 3-(1*H*-benzo[*d*]imidazol-2-yl)-2-(2-chlorophenyl)thiazolidin-4-one (4f)**


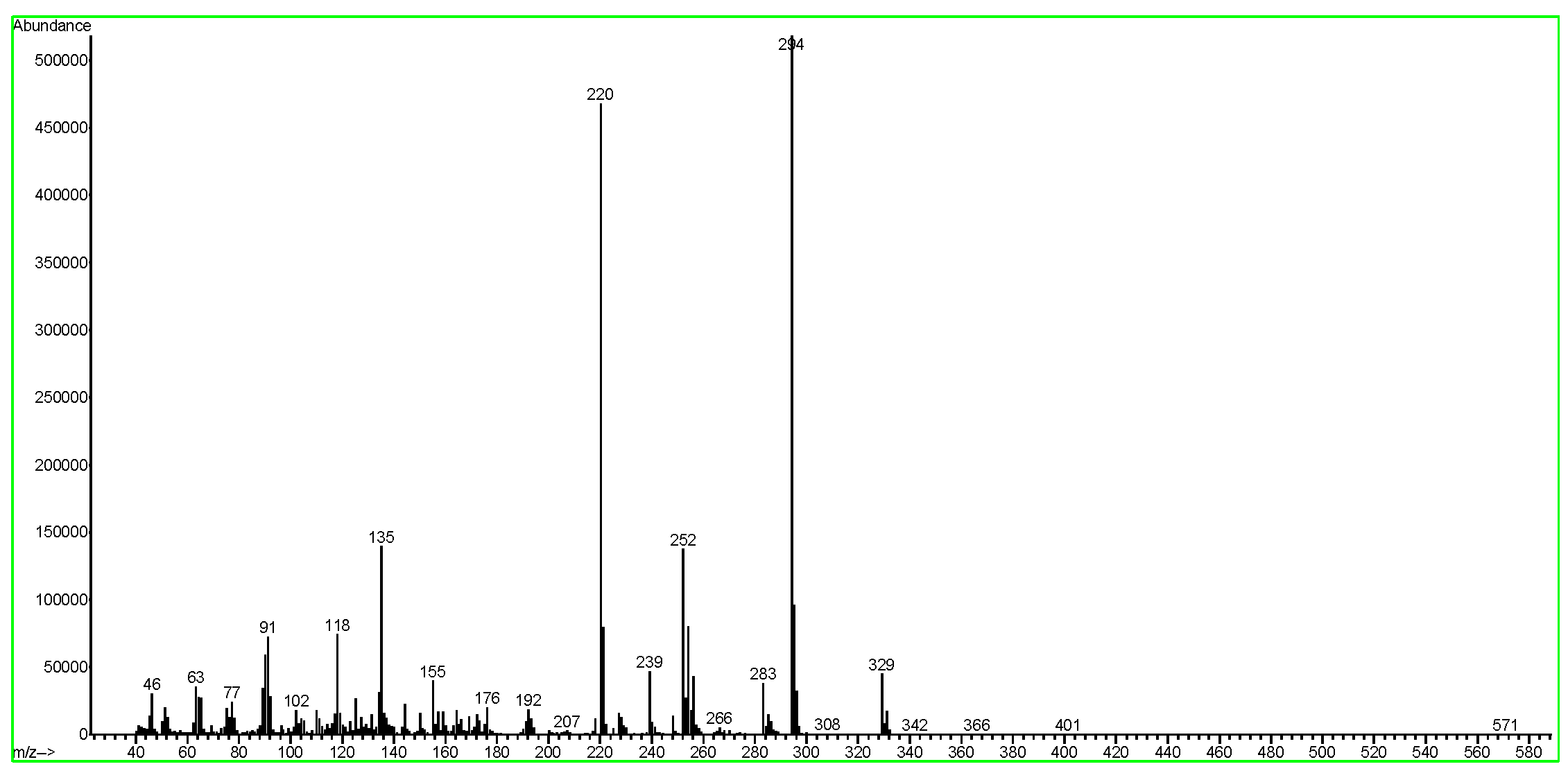


**Fig. S19. The ^1^H NMR spectrum of 3-(1*H*-benzo[*d*]imidazol-2-yl)-2-(4-methoxyphenyl)thiazolidin-4-one (4g)**


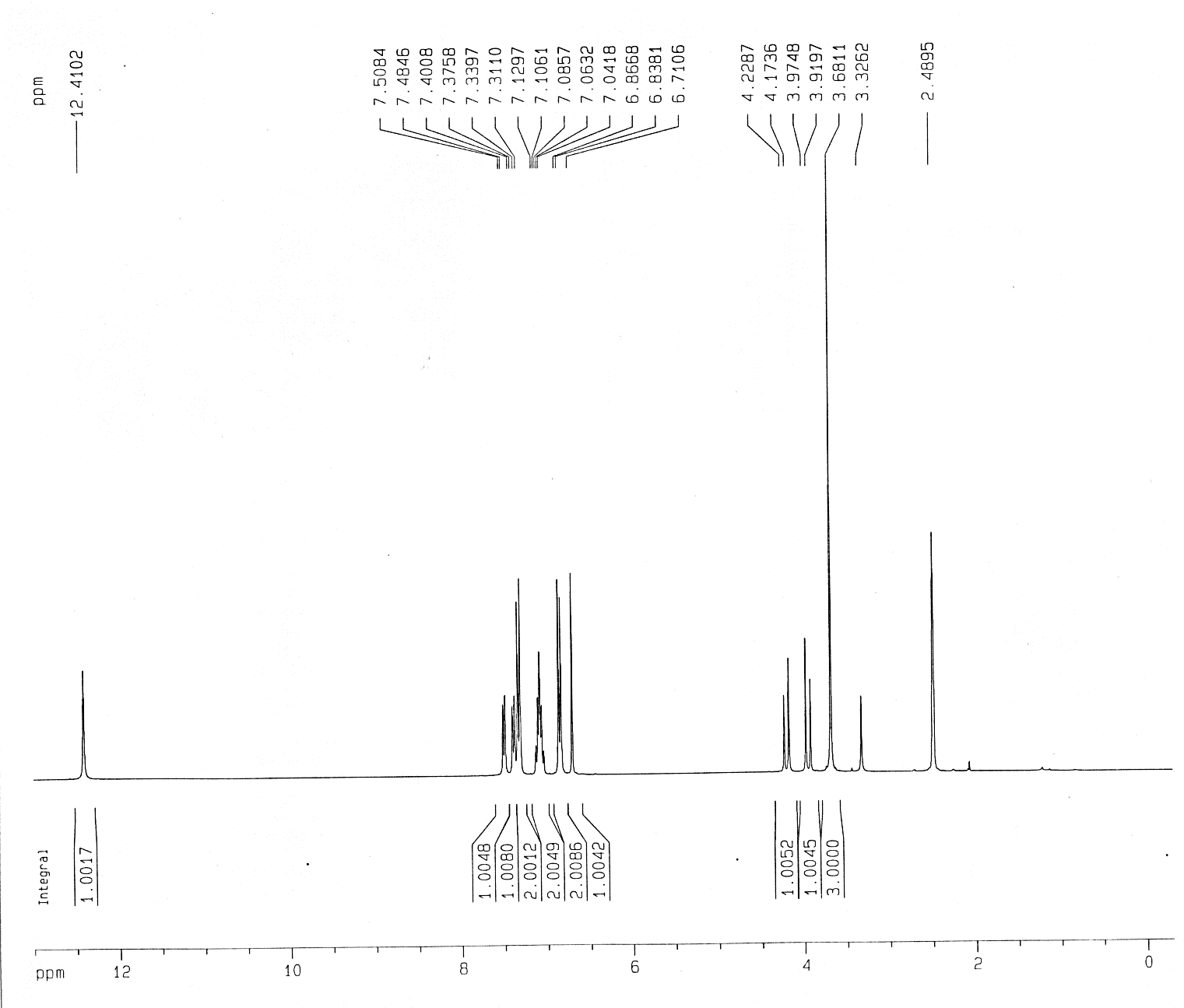


**Fig. S20. The ^13^C NMR spectrum of 3-(1*H*-benzo[*d*]imidazol-2-yl)-2-(4-methoxyphenyl)thiazolidin-4-one (4g)**


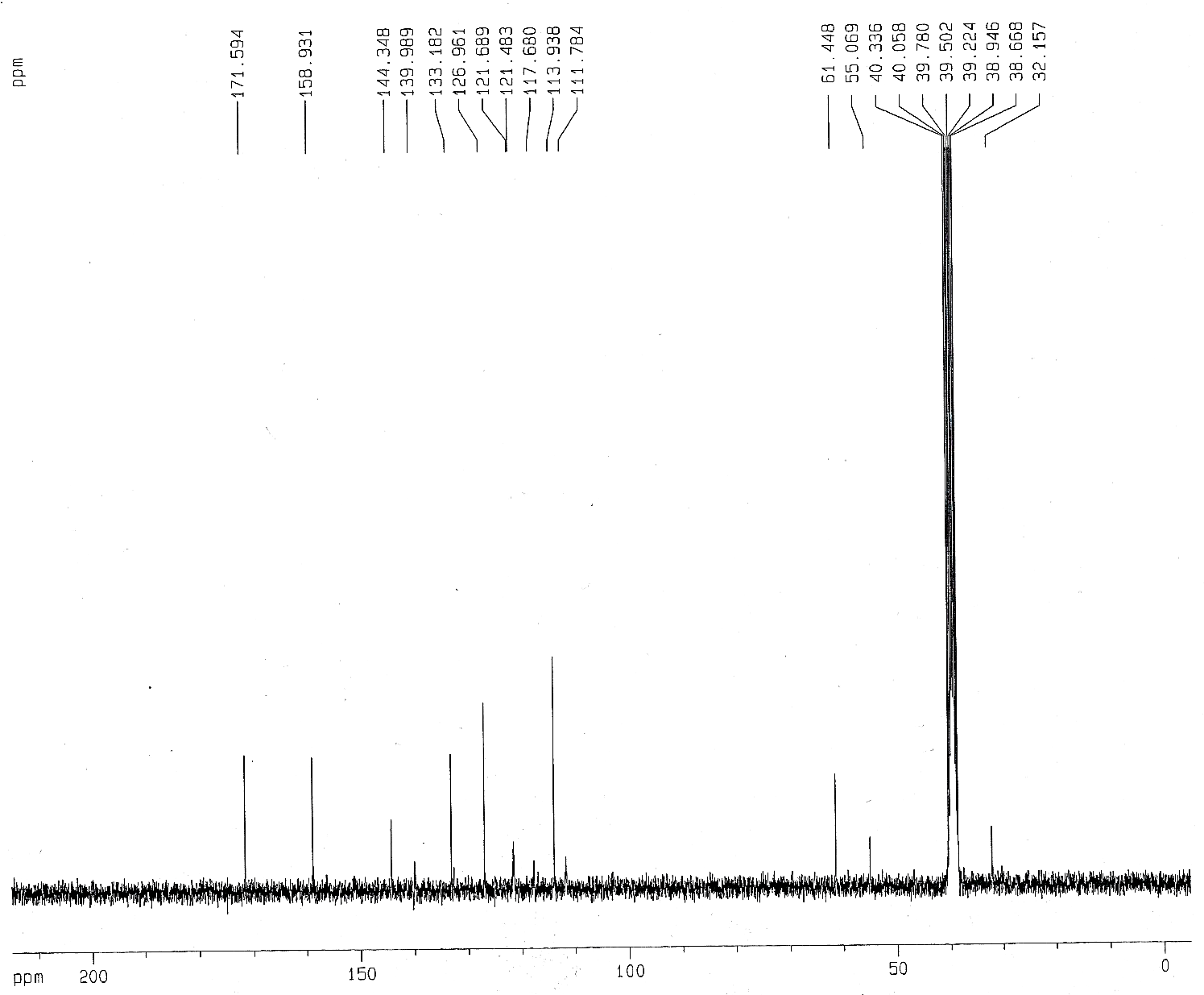


**Fig. S21. The Mass spectrum of 3-(1*H*-benzo[*d*]imidazol-2-yl)-2-(4-methoxyphenyl)thiazolidin-4-one (4g)**

**Fig. S22. The ^1^H NMR spectrum of 3-(1*H*-benzo[*d*]imidazol-2-yl)-2-(*p*-tolyl) thiazolidin-4-one (4h)**

**
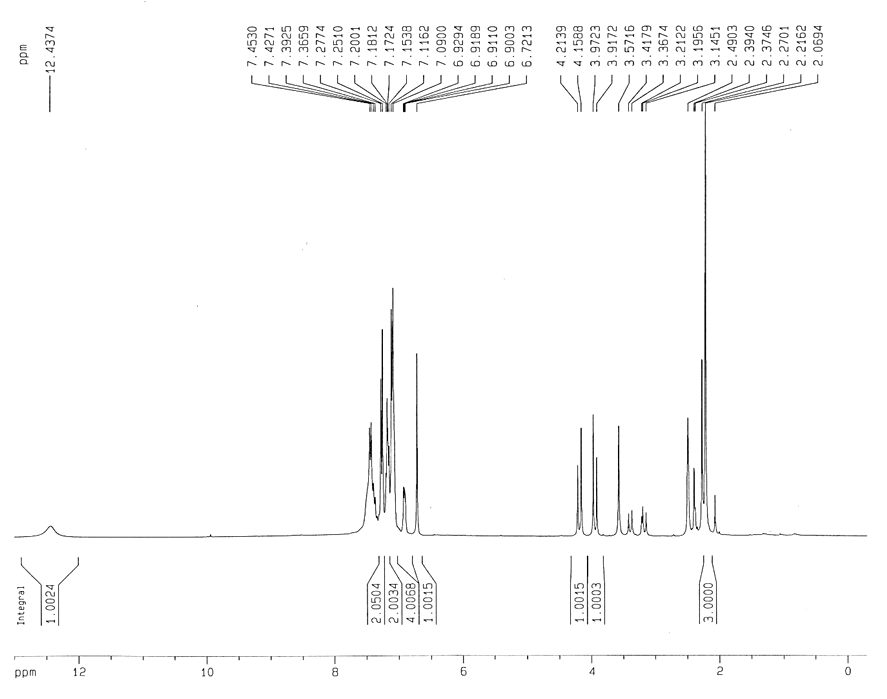
**

**Fig. S23. The ^13^C NMR spectrum of** **3-(1*H*-benzo[*d*]imidazol-2-yl)-2-(*p*-tolyl) thiazolidin-4-one (4h)**


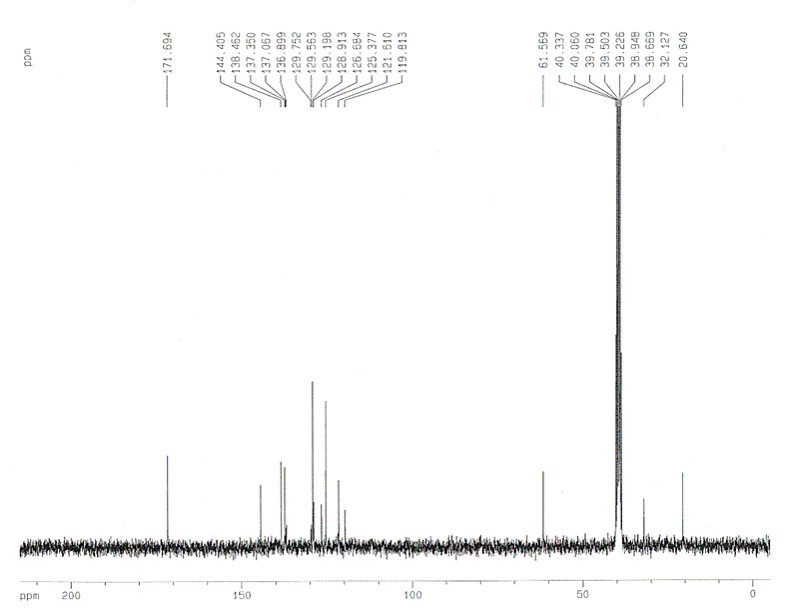


**Fig. S24. The Mass spectrum of** **3-(1*H*-benzo[*d*]imidazol-2-yl)-2-(*p*-tolyl) thiazolidin-4-one (4h)**

**Fig. S25. The ^1^H NMR spectrum of 3-(1*H*-benzo[*d*]imidazol-2-yl)-2-(3,4-dimethoxyphenyl)thiazolidin-4-one (4i)**


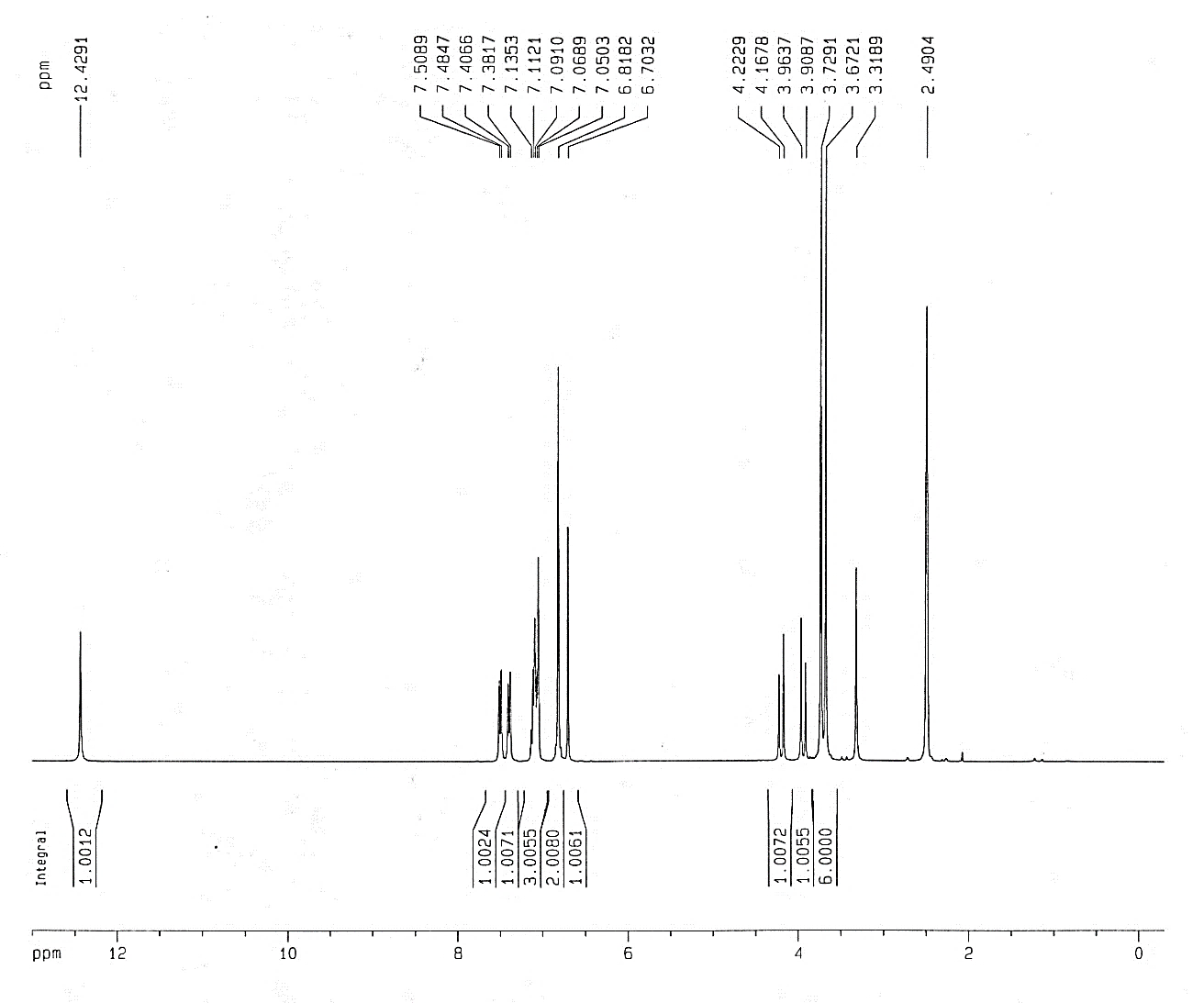


**Fig. S26. The ^13^C NMR spectrum of 3-(1*H*-benzo[*d*]imidazol-2-yl)-2-(3,4-dimethoxyphenyl)thiazolidin-4-one (4i)**


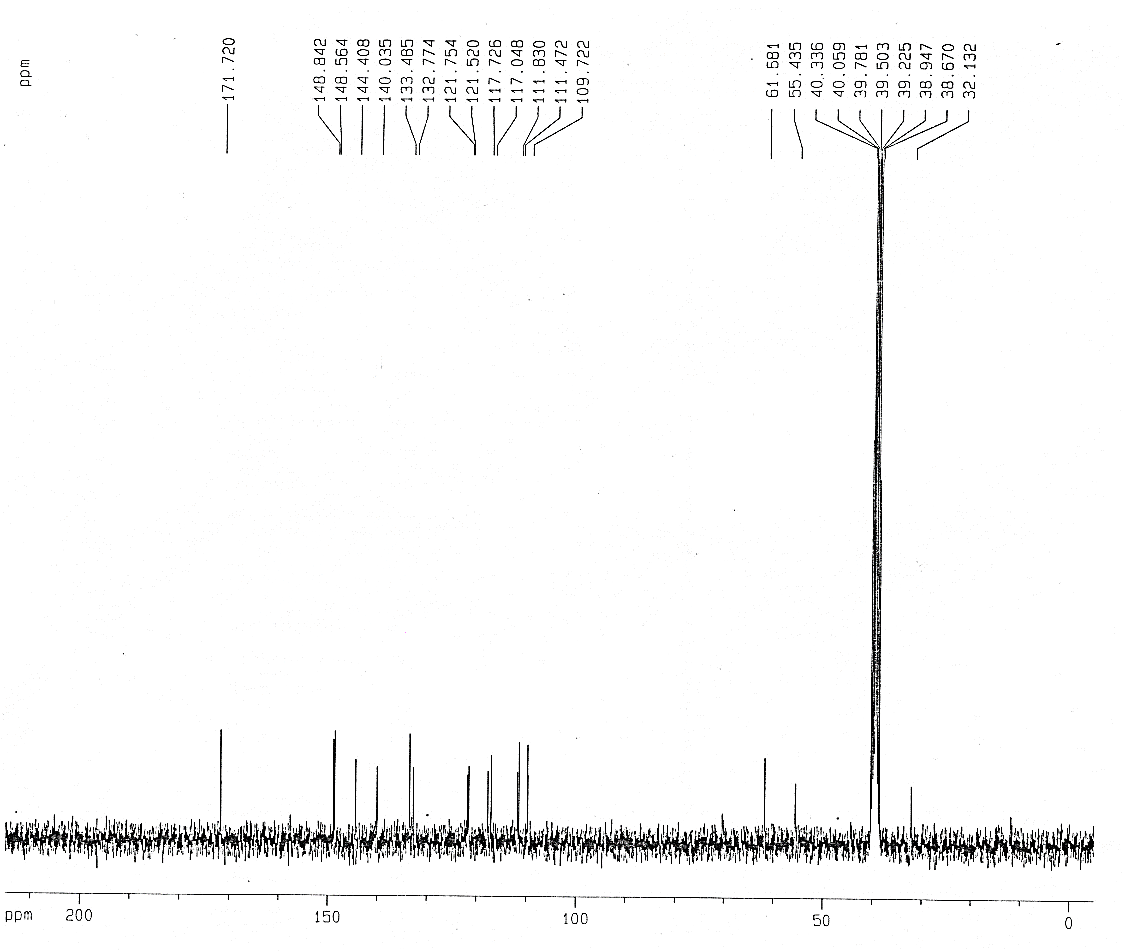


**Fig. S27. The Mass spectrum of 3-(1*H*-benzo[*d*]imidazol-2-yl)-2-(3,4-dimethoxyphenyl)thiazolidin-4-one (4i)**

**Fig. S28. The ^1^H NMR spectrum of 3-(Benzo[*d*]thiazol-2-yl)-2-(4-nitrophenyl)thiazolidin-4-one (4j)**


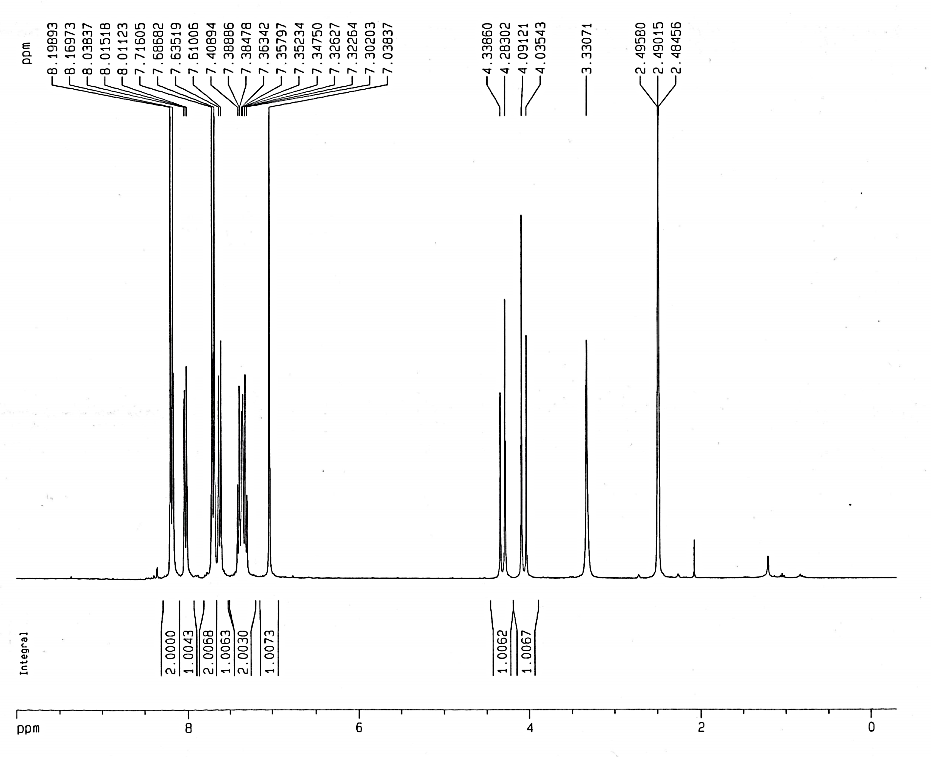


**Fig. S29. The ^13^C NMR spectrum of 3-(Benzo[*d*]thiazol-2-yl)-2-(4-nitrophenyl)thiazolidin-4-one (4j)**

**
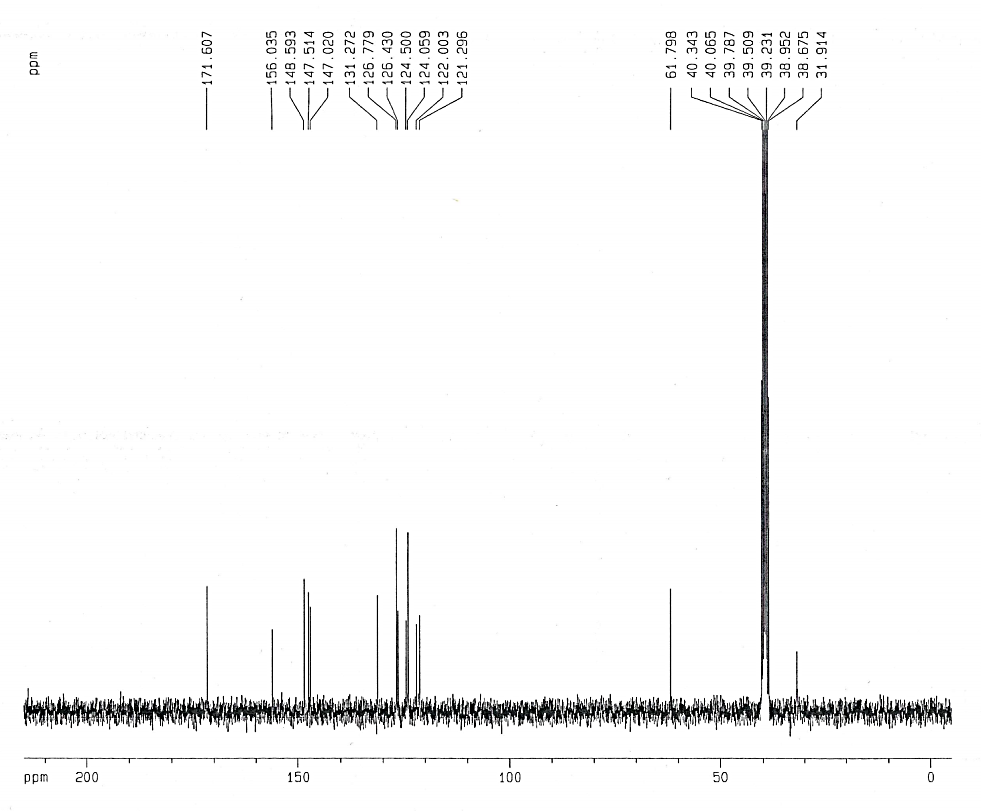
**

**Fig. S30. The Mass spectrum of 3-(Benzo[*d*]thiazol-2-yl)-2-(4-nitrophenyl)thiazolidin-4-one (4j)**

**
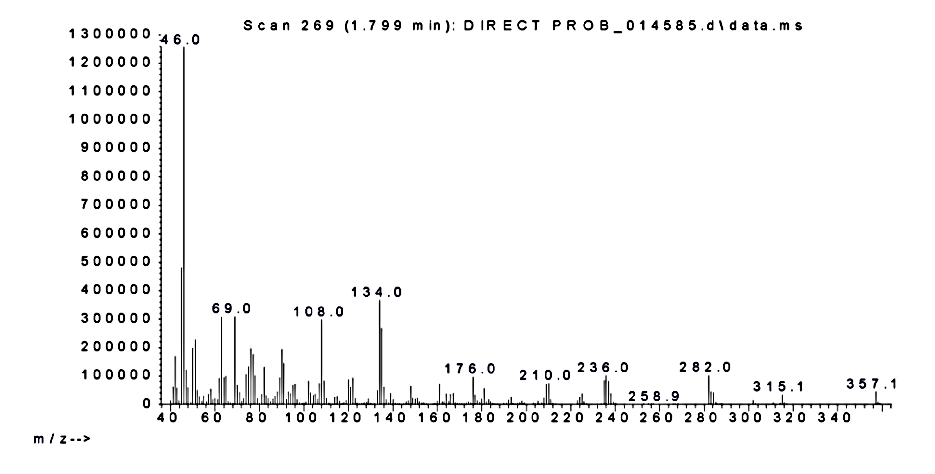
**

**Fig. S31. The ^1^H NMR spectrum of 3-(Benzo[*d*]thiazol-2-yl)-2-(3-nitrophenyl)thiazolidin-4-one (4k)**


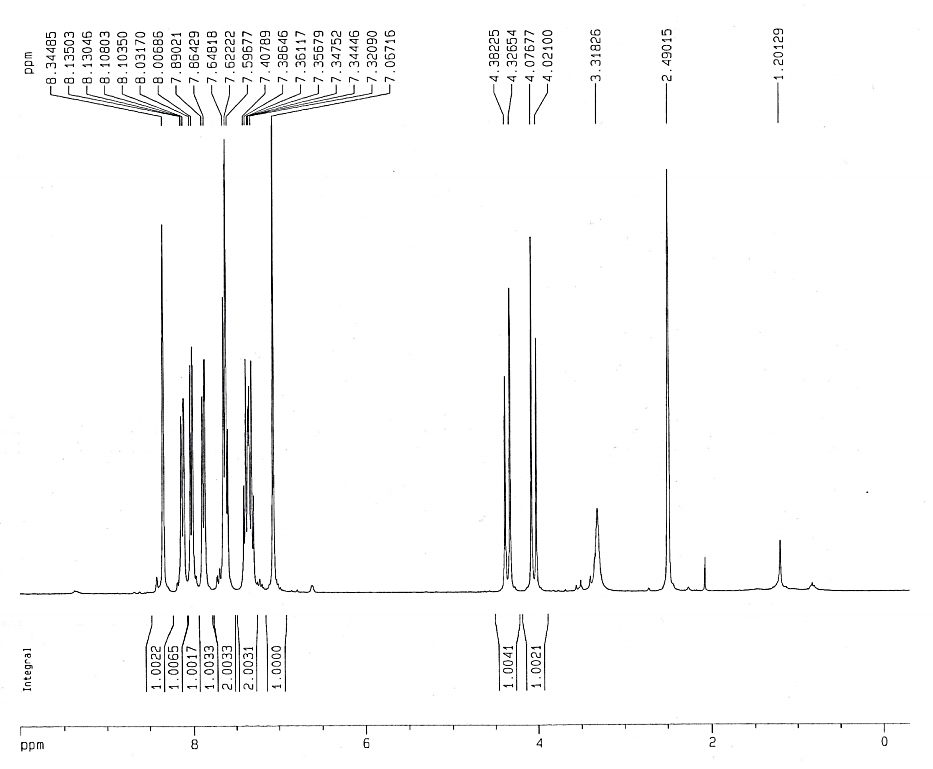


**Fig. S32. The ^13^C NMR spectrum of 3-(Benzo[*d*]thiazol-2-yl)-2-(3-nitrophenyl)thiazolidin-4-one (4k)**

**
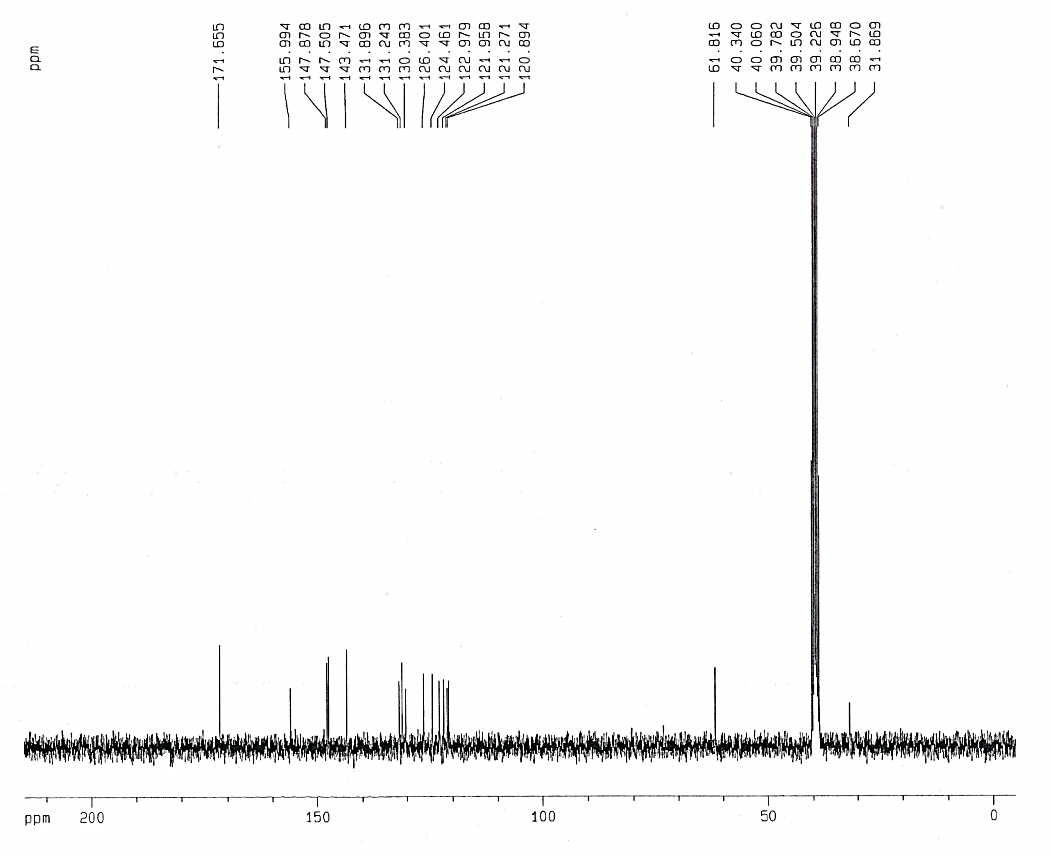
**

**Fig. S33. The Mass spectrum of 3-(Benzo[*d*]thiazol-2-yl)-2-(3-nitrophenyl)thiazolidin-4-one (4k)**

**
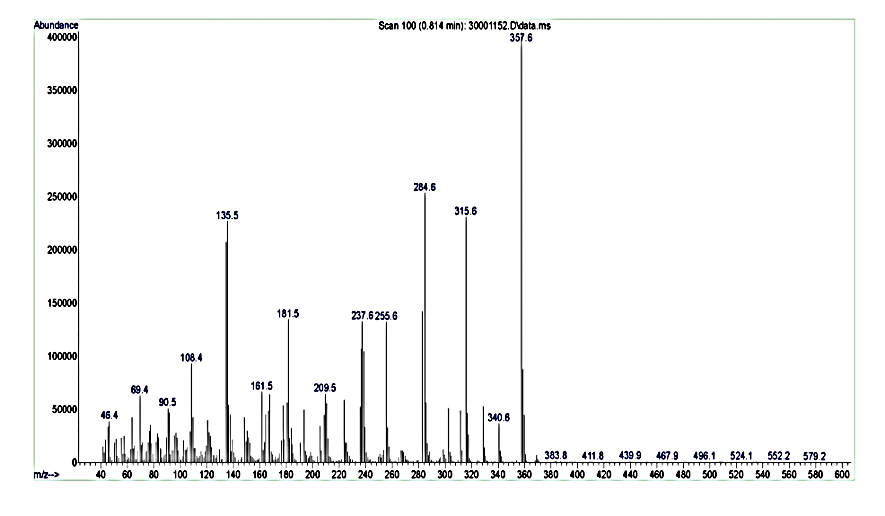
**

**Fig. S34. The ^1^H NMR spectrum of 3-(Benzo[*d*]thiazol-2-yl)-2-(4-methylphenyl)thiazolidin-4-one (4l)**

**
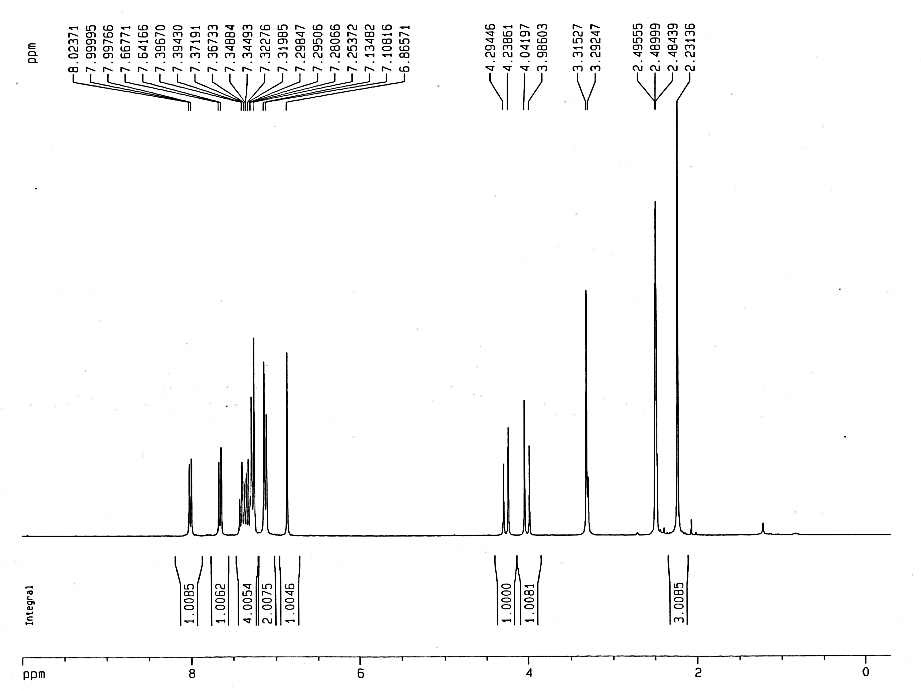
**

**Fig. S35. The ^13^C NMR spectrum of 3-(Benzo[*d*]thiazol-2-yl)-2-(4-methylphenyl)thiazolidin-4-one (4l)**


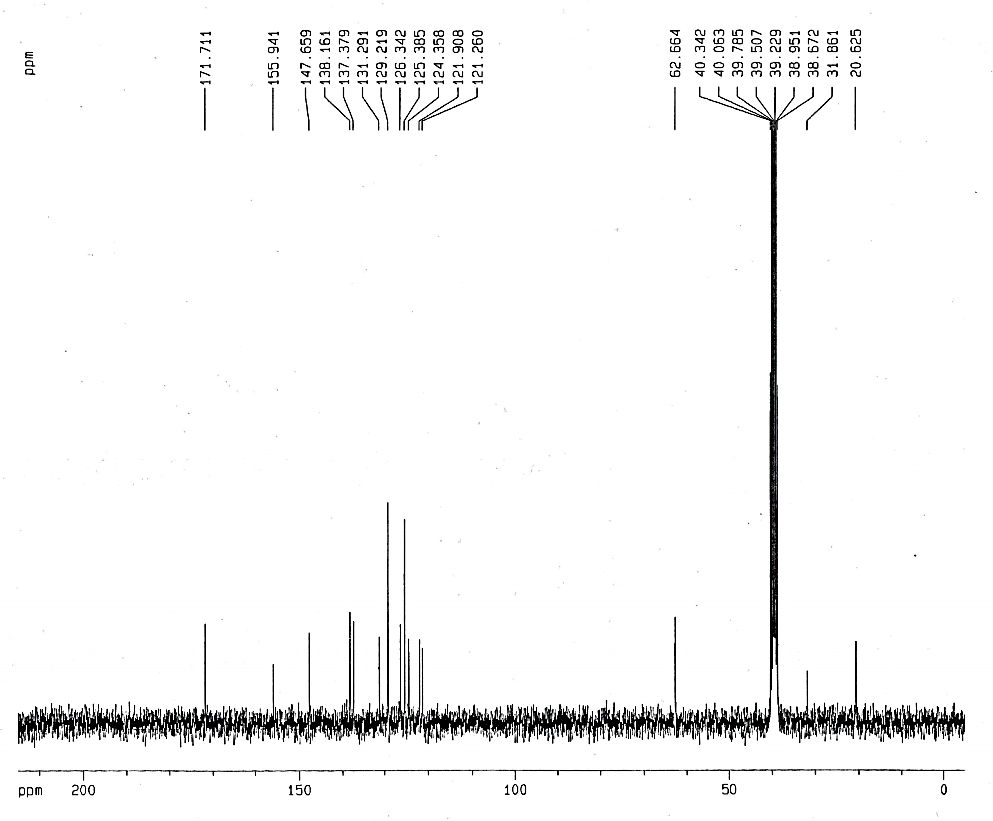


**Fig. S36. The Mass spectrum of 3-(Benzo[*d*]thiazol-2-yl)-2-(4-methylphenyl)thiazolidin-4-one (4l)**

**
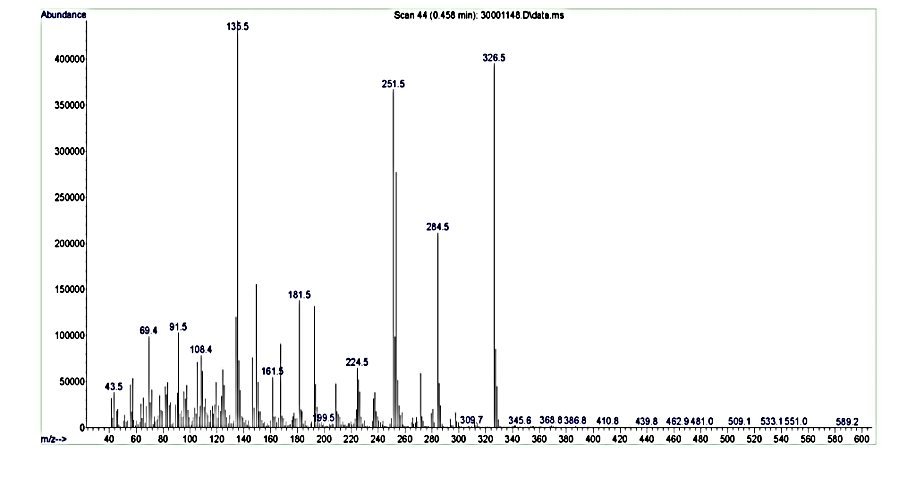
**

**Fig. S37. The ^1^H NMR spectrum of 3-(Benzo[*d*]thiazol-2-yl)-2-(4-bromophenyl)thiazolidin-4-one (4m)**


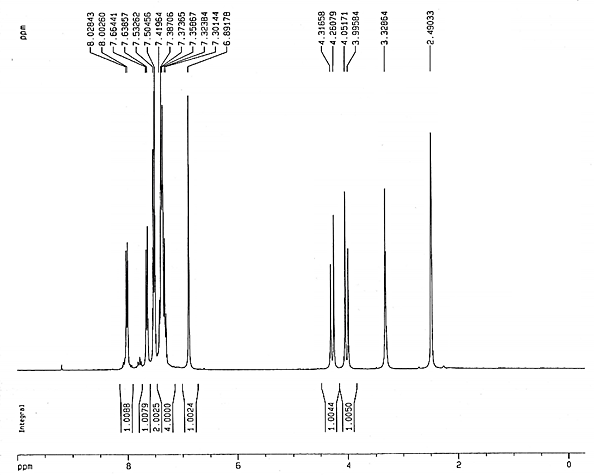


**Fig. S38. The ^13^C NMR spectrum of 3-(Benzo[*d*]thiazol-2-yl)-2-(4-bromophenyl)thiazolidin-4-one (4m)**

**
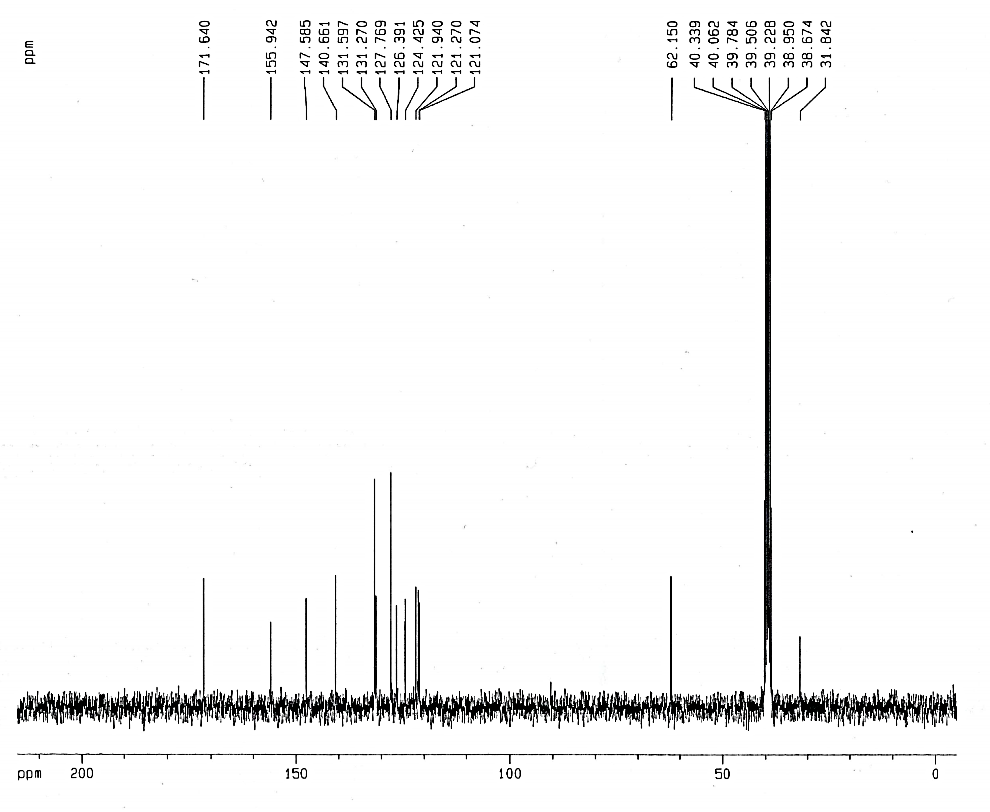
**

**Fig. S39. The Mass spectrum of 3-(Benzo[*d*]thiazol-2-yl)-2-(4-bromophenyl)thiazolidin-4-one (4m)**

**
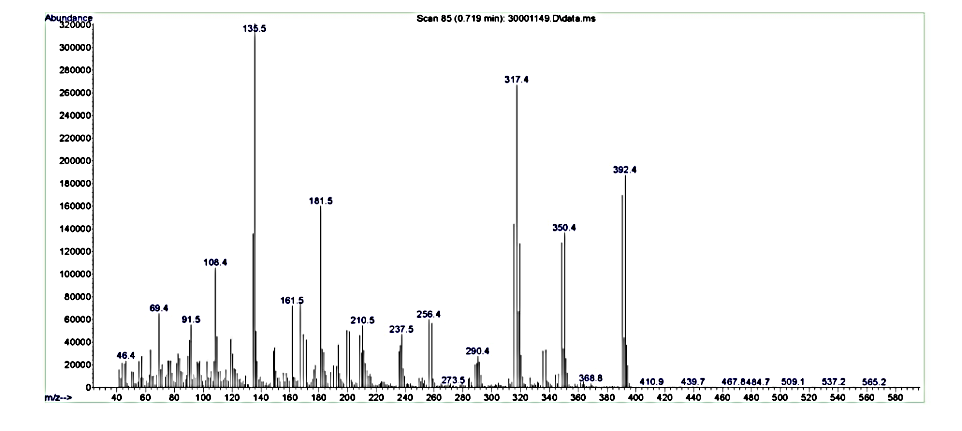
**

**Fig. S40. The ^1^H NMR spectrum of 3-(Benzo[*d*]thiazol-2-yl)-2-(4-methoxyphenyl)thiazolidin-4-one (4n)**

**
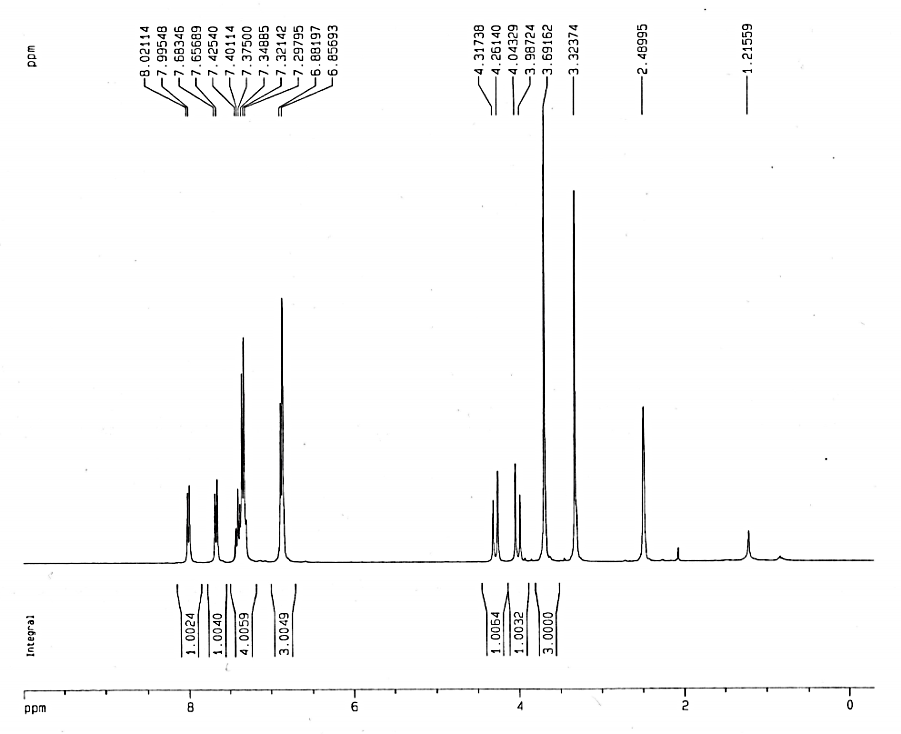
**

**Fig. S41. The ^13^C NMR spectrum of 3-(Benzo[*d*]thiazol-2-yl)-2-(4-methoxyphenyl)thiazolidin-4-one (4n)**

**
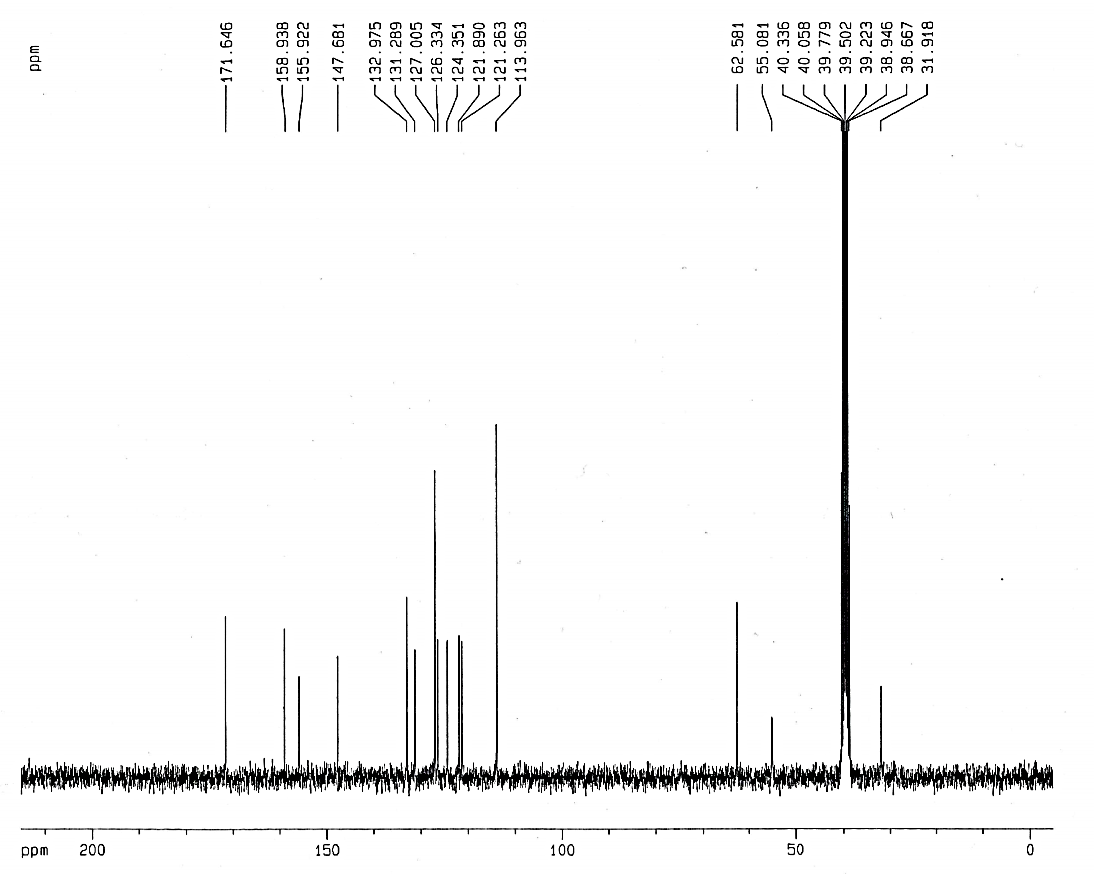
**

**Fig. S42. The Mass spectrum of 3-(Benzo[*d*]thiazol-2-yl)-2-(4-methoxyphenyl)thiazolidin-4-one (4n)**

**
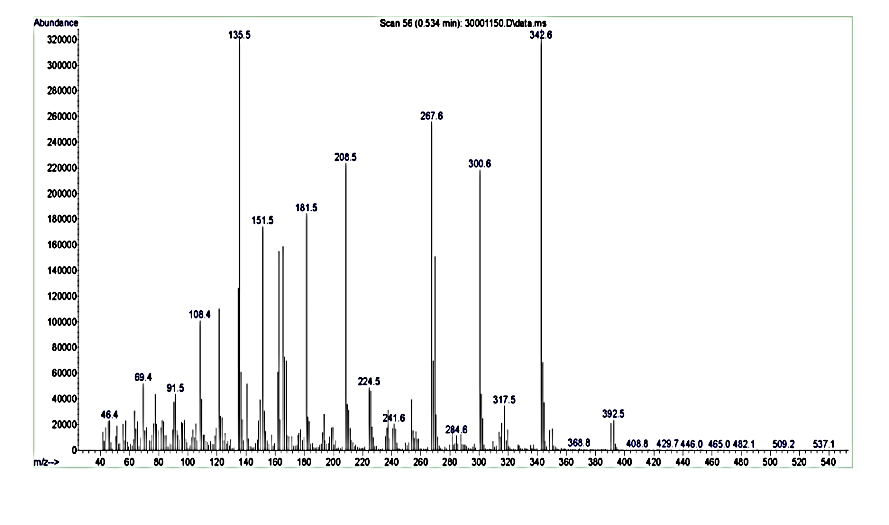
**
